# Integrative transcriptomic and proteomic analyses reveal a positive role of BES1 in salt tolerance in *Arabidopsis*

**DOI:** 10.3389/fpls.2023.1034393

**Published:** 2023-03-01

**Authors:** Lei Feng, Yan Li, Yu-Ling Zhou, Guang-Hua Meng, Zhao-Lin Ji, Wen-Hui Lin, Jun-Xian He

**Affiliations:** ^1^ School of Life Sciences and State Key Laboratory of Agrobiotechnology, The Chinese University of Hong Kong, Sha Tin, New Territories, Hong Kong, China; ^2^ State Key Laboratory of Subtropical Silviculture, Zhejiang Agriculture and Forestry University, Hangzhou, China; ^3^ College of Plant Protection, Yangzhou University, Yangzhou, China; ^4^ The Joint International Research Laboratory of Metabolic and Developmental Sciences, School of Life Sciences and Biotechnology, Shanghai Jiao Tong University, Shanghai, China; ^5^ Shanghai Collaborative Innovation Center of Agri-Seeds, Joint Center for Single Cell Biology, Shanghai Jiao Tong University, Shanghai, China

**Keywords:** brassinosteroids, salt tolerance, transcriptomics, proteomics, *Arabidopsis thaliana*, BES1

## Abstract

**Introduction:**

Salt stress is a major environmental factor limiting plant growth and development. Previous studies have indicated that the steroidal hormones—brassinosteroids (BRs) are important regulators of plant responses to salt stress. However, the underlying molecular mechanisms have not been fully understood.

**Methods:**

(1) Phenotypic analysis of *bes1-D, BES1-RNAi* and their wild-type (Col-0) under salt treatments with different concentrations of NaCl. (2) Transcriptomic and proteomic profiling of BES1-regulated genes and proteins under salt treatment; (3) qRT-PCR validation of selected BES1-regulated genes under salt stress; (4) Transient transcriptional assay of BES1 regulation on its putative target genes in *Arabidopsis* protoplasts; (5) Electrophoresis Mobility Shift Assay (EMSA) of BES1 binding with its potential target genes.

**Results and Discussion:**

Phenotypic analysis indicated that *bes1-D*, a gain-of-function mutant of the BR-regulated transcription factor BES1 in *Arabidopsis* showed better salt tolerance than the wild-type plant, while a BES1 RNA interference (*BES1-RNAi*) line was more sensitive to salt stress. Global gene expression profiling and time series clustering analyses identified a total of 1,170 genes whose expression was boosted in *bes1-D* under salt stress. Further GO enrichment and gene functional network analyses identified several key modules that are regulated by BES1 and most sensitive to salt stress perturbations, including stress response, response to ABA and ROS, flavonoid biosynthesis and transmembrane transport. A comparative proteomic analysis performed under the same stress conditions supported the results from the transcriptome analysis. In addition, transient gene transcription assays in *Arabidopsis* protoplasts and *in vitro* DNA binding assays verified that BES1 regulates the expression of some ion transporter genes directly and indirectly. Taken together, our results support a positive role of BES1 in plant salt tolerance.

## Introduction

Salt stress adversely affects plant growth and reduces crop yields. It was reported that salt-affected soil accounted for around 10% of irrigated land (Shahid et al., 2018). In order to adapt to salt stress, plants have evolved some salt response mechanisms. The Salt Overly Sensitive (SOS) pathway plays important roles in perceiving the salt signal and export excess Na^+^ out of cytoplasm, thus conferring salt tolerance to plants (Zhu, 2002). The extracellular Na^+^ is first sensed by glycosyl inositol phosphoryl ceramides (GIPCs), a class of glycosylated sphingolipids in plasma membrane, which bind Na^+^ and trigger a quick increase in cytoplasmic Ca^2+^ concentration ([Ca^2+^]) through activating the gated Ca^2+^ influx channels (Jiang et al., 2019b). These cytosolic Ca^2+^ signals then activate the SOS pathway, in which the calcineurin B-like (CBL) proteins SOS3/CBL4 or Calcium Binding Protein 8 (SCaBP8) receive the [Ca^2+^] signals (Quan et al., 2007; Quintero et al., 2011) and recruits the CBL-interacting protein kinase SOS2/CIPK24 to plasma membrane to activate SOS1, a Na^+^/H^+^ antiporter, through phosphorylation. The activated SOS1 will mediate Na^+^ efflux and help intracellular ion homeostasis (Shi et al., 2000; Guo et al., 2001). The SOS3-SOS2 complex also positively regulate NHX, a vacuolar Na^+^/H^+^ exchanger, which transports Na^+^ from the cytoplasm to the vacuole (Brini and Masmoudi, 2012). Furthermore, the Na^+^ transporter HKT1 (High-Affinity K^+^ Transporter 1), which mainly expresses in the root xylem parenchyma cells, is believed to participate in long-distance transportation and distribution of Na^+^ and K^+^ in plants (Mäser et al., 2002; Sunarpi et al., 2005; Deinlein et al., 2014). Recently, two phosphatases PP2C.D6 and PP2C.D7 were found to be able to inactive SOS1 through dephosphorylation under nonstress conditions, however, under salt stress conditions these phosphatases are inhibited by SCaBP8, therefore obviating their inhibition on SOS1 (Fu et al., 2022). Besides causing ionic stress, excess salt also causes osmotic stress and oxidative stress (Yang and Guo, 2018). The osmotic stress is mediated by ABA-dependent and independent signaling pathways and these signal cascades further regulate downstream transcriptional gene expression (Soma et al., 2017). Salt stress often induces rapid production of reactive oxygen species (ROS), which may result in oxidation stress. ROSs also function as signaling molecules to regulate ion homeostasis through MAPK cascades (Pitzschke et al., 2009). ROSs are toxic and must be kept at suitable levels. To reduce excess ROS accumulation, plants have evolved both enzymatic and nonenzymatic scavenging systems to mitigate ROS-induced damages (del Río and López-Huertas, 2016).

BRs are a group of steroid phytohormones that promote plant growth and developments. BR signal is perceived by the cell-surface receptor kinase BRI1 (brassinosteroid insensitive 1) and its coreceptor BAK1 (BRI1-associated receptor kinase 1) (Nam and Li, 2002). BR binding activates BRI1’s kinase activity, leading to phosphorylation and activation of BSK1 and CDG1, two cytoplasmic receptor-like kinases. BSK1 and CDG1 mediate the signal transduction to BSU1 (BRI1 suppressor 1), a PP1-like phosphatase through phosphorylation (Kim et al., 2011). BSU1 then dephosphorylates and inactivates a downstream GSK3-like kinase BIN2 (Kim et al., 2009), which negatively regulates BR signaling by phosphorylating and destabilizing the transcription factors BZR1 (brassinazole resistant 1) and its paralogue BES1 (BRI1-EMS-suppressor 1, also named as BZR2) (He et al., 2002; Yin et al., 2002; Zhao et al., 2002). Hence, inactivation of BIN2 during BR signal transduction will release the transcriptional activity of BZR1 and BES1. The dephosphorylated BRZ1 and BES1 subsequently translocate into the nucleus and regulate the expression of their target genes by binding to the BRRE or E-box motifs in their promoters (Wang et al., 2002; Li et al., 2018; Clark et al., 2021). Genome-wide target gene profiling experiments revealed that BZR1 and BES1 target thousands of genes involved in multiple aspects of plant development (Sun et al., 2010; Yu et al., 2011; Nolan et al., 2017b)

BIN2 and BZR1/BES1 have been shown to act as mediators of crosstalk between BR signaling and other pathways. For example, BIN2 and BES1 were reported to interact with RD26 (responsive to desiccation 26) to coordinate BR- and ABA-regulated drought resistance. Under normal conditions, RD26 is transcriptionally repressed by BES1, however, under drought stress conditions, RD26 is induced to antagonize BES1’s transcriptional activity through direct interaction with BES1 and to repress plant growth, which is beneficial to drought survival of plants (Ye et al., 2017). The same authors also found that ABI1, a member of the 2C class of protein phosphatases (PP2Cs, negative regulators of ABA signaling), could inhibit BIN2’s kinase activity *via* dephosphorylation under normal conditions. Drought stress relieves ABI1 inhibition of BIN2, and the activated BIN2 strengthens drought tolerance through phosphorylating and stabilizing RD26 (Jiang et al., 2019a). Another parallel study also found that under drought conditions BIN2 can phosphorylate and activate SnRK2 kinase in ABA signaling, thus promoting ABA responsive gene expression (Wang et al., 2018). Therefore, BR represses ABA responsive genes through BIN2 under normal conditions, thus promoting plant growth (Cai et al., 2014; Hu and Yu, 2014; Wang et al., 2018). These studies confirmed an antagonism relationship between BR and ABA and provided a mechanism for balancing plant growth and drought survival (Nolan et al., 2017a; Nolan et al., 2019; Kono and Yin, 2020). In addition to drought, BZR1 and BES1 are also involved in plant responses to freezing (Li et al., 2017), heat (Yin et al., 2018; Albertos et al., 2022; Chen et al., 2022) and salinity stress. In the case of salt stress, BIN2 was found to act as a molecular switch to help plant recovery after non-lethal salt stress in *Arabidopsis*. BIN2 is guided to plasma membrane by SOS3 and SCaBP8, where it inhibits SOS2 through phosphorylation, therefore negatively regulating plant salt tolerance (Li et al., 2020). In addition, *SlBZR1*, a *BZR1* homologue in tomato, was found to enhance salt tolerance in both tomato and *Arabidopsis*, and upregulate the expression of several stress responsive genes (i.e., *SlAREB1, SlDREB1, SlRD29, SlERF1, SlCAT2, SlAPX2*) (Jia et al., 2021). Recently, *VvBES1-3, a BES1* homologue from grape, was found to improve salt tolerance in transgenic *Arabidopsis* (Cao et al., 2023). Despite these interesting findings, it remains largely unknown for the mechanisms by which BZR1/BES1 mediate salt stress responses in plants.

In this study, we evaluated the role of BES1 in *Arabidopsis* salt responses at the genome level by using the *bes1-D* and *BES1-RNAi* mutant lines. Based on the phenotypes observed, we performed transcriptome sequencing to explore the molecular basis of BES1-induced salt resistance. Time series clustering analysis identified gene clusters specifically changed in *bes1-D* under salt stress. GO enrichment network analysis suggested a few key modules regulated by BES1. Further proteomic analysis and qRT-PCR assays were used to deepen our understanding of the salt stress responses in *bes1-D*. Finally, we selected several potential target genes of BES1 in regulating plant salt resistance and validated them by transient transcription assays and *in vitro* DNA binding assay (EMSA). This study provided new insights to the role of BR in plant responses to salt stress.

## Materials and methods

### Plant materials and treatment

*A. thaliana* seeds (Col-0, *bes1-D*, *BES1-RNAi*, and BES1-FLAG) were surface sterilized for 6 h using the vapor generated by hydrochloric acid and sodium hypochlorite (Lindsey et al., 2017). After sterilization, seeds were sown in petri dishes (10 × 1.5 cm) containing half-strength ½ Murashige and Skoog (MS) medium with 0.8% agarose and vernalized at 4°C in the dark for 2 d. Then, these petri dishes were placed in a plant growth chamber at temperature of 23°C day/21°C night, photoperiod of 12 h light/12 h dark, with photosynthetic active radiation (PAR) of 100 μmol·m^−2^·s^−1^, and relative humidity of 70%. Six days later, forty seedlings of each material (Col-0, *bes1-D*, and *BES1-RNAi*) were transferred into new petri dishes containing ½ MS solid medium with different concentrations of NaCl (0, 100, 150, and 200 mM). At the treatment of 2, 4, and 6 days, their phenotypes were photographed, and survival rates were calculated as the ratio of number of surviving seedlings to total seedlings. The same samples were also used for determination of relative electrolyte leakage (REL). The experiments were repeated three times. ANOVA and LSD *post-hoc* analysis in SPSS were used for statistical analysis of change significances.

### Measurement of relative electrolyte leakage

Membrane relative permeability or electrical conductivity was measured according to Cao et al. (2007). Each sample (Col-0, *bes1-D* or *BES1-RNAi*) collected at different time points of the treatment was put into a 50-mL Falcon tube containing 30 mL of milli-Q water and left for 20 h, and their electrical conductivity (L1) were measured by a conductivity detector DDS-11A (Kangyi, China), respectively. Next, these tissue samples were killed at 121°C in a heating chamber for 30 minutes, and their electrical conductivity (L2) were determined sequentially. Relative electrolyte leakage (REL) (%) = (L1 − electrical conductivity of milli-Q water)/(L2 − electrical conductivity of milli-Q water). The experiments were repeated three times.

### RNA extraction, RNA sequencing, and data analysis

Seedlings of *bes1-D* and Col-0 treated with 150 mM NaCl or normal ½ MS medium were collected at two time-points (2 days and 4 days), respectively. Three independent biological repeats were prepared for each time point and the total sample size is 24 (**Supplementary Table S1**). Total RNA was extracted from these tissues by the Trizol reagent (Takara, China), dissolved in DEPC-treated water and stored at –80°C until use. RNA quality was measured by NanoDrop 2000 spectrometer and Agilent 2100 Bioanalyzer, respectively. Strand-specific RNA-Seq libraries were constructed according to Horvath et al. (2015) with the Illumina TruSeq RNA preparation kit (Illumina Inc, USA). After second strand cDNA synthesis, cDNA was fragmented, ligated with adapters, and amplified by PCR. Quantitated cDNA libraries were mixed and loaded on a Flow Cell. The cDNA libraries were sequenced on an Illumina HiSeq4000™ platform with pair-end 100 bp mode (BGI, Hong Kong). Total raw data of more than 138 Gb was generated, with an average of 5.77 Gb sequence data for each sample (**Supplementary Table S2**).

Transcriptome data was analyzed by using the protocol of Pertea et al. (2016). Briefly, clean data (high-quality reads) were mapped to *Arabidopsis* reference genome by using HISAT2 v2.2.1 with parameters “–rf –trim5 10” (Kim et al., 2019). The reference sequences of *A. thaliana* genome (TAIR10 release, chromosome length = 119,146,348 bp) was downloaded from www.arabidopsis.org. FPKM (Fragments per kilobase per million mapped reads) values of each gene were calculated by using StringTie v2.1.4 with parameters “-e -B” (Pertea et al., 2015). The full gene dataset of *A. thaliana* (*Araport11_GFF3_genes_transposons.201606.gff*) was from TAIR database version Araport11, which contains 37,866 gene entries. Differentially expressed genes (DEGs) between experimental and control groups were analyzed by using *Ballgown* package in Bioconductor with *stattest* function whose parameters were set as: feature=‘gene’, getFC=TRUE, meas=‘FPKM’ (Frazee et al., 2015). Threshold of significant DEGs was defined as below criterial: fold change (absolute value) > 1.5 and *P* value < 0.05. The RNA sequencing data have been deposited in NCBI Sequence Read Archive database (SRA, http://www.ncbi.nlm.nih.gov/sra) with SRA accession number PRJNA882207.

### Time series clustering analysis of gene expression

RNA read counts for each gene were calculated by *htseq-count* v0.13.5 with parameters “-m union” (Anders et al., 2015). The general feature file (*Araport11_GFF3_genes_transposons.201606.gff*) was modified to exclude transposable elements and microRNAs for read counting. Time series clustering analysis was performed by using *TCseq* packages v1.20.0 in Bioconductor. High quality genic regions with read counts > 10 in at least two samples were kept for downstream analysis (filter.type = “raw”, filter.value = 10, samplePassfilter = 2). Gene counts were further transformed into a time-course table for clustering analysis (value = “expression”, lib.norm = TRUE, norm.method = “rpkm”, filter = TRUE, pvalue.threshold = 0.05, abs.fold = 1.5). Fuzzy c-means algorithm was adopted for clustering. Gene expression was normalized by Z-score transformation (algo = “cm”, k = 6, standardize = TRUE).

### Gene ontology enrichment and network analysis

Gene Ontology (GO) describes biological process (BP), molecular function (MF), or cellular component (CC) for genes. GO enrichment analyses for DEGs or differentially expressed proteins (DEPs) were performed by using BiNGO plugin in Cytoscape v3.9.1 (Maere et al., 2005). In this study, only “biological process” was selected for the enrichment analysis. *P*-values were calculated by hypergeometric test and their false discovery rates (FDRs) were adjusted by Benjamini-Hochberg method. All unique genes with biological process information (22,304 genes) in *A. thaliana* were used as the reference set.

GO network analyses were performed by using ClueGO function in CytoScape, as described by Zeng et al. (2021). The biological process dataset of *A. thaliana* which contains 28,035 genes were chosen as background (version 21.03.2022). *P*-values were calculated by two-side hypergeometric test and their FDRs were adjusted by Bonferroni–Holm method. GO items belong to Level 3–7 were selected for network construction. Term-term interactions and GO clusters were inferred by Kappa score (Bindea et al., 2009).

### Protein extraction for proteomic analysis

The same samples from the 2-day treatment group for transcriptome sequencing (**Supplementary Table S1**) were used for proteomic assays. Total proteins were extracted from *Arabidopsis* seedlings according to Wu et al. (2020). In brief, plant tissues were mixed with 5% PVPP (plolyvinylpyrrolidone) powder and lysis buffer (7 M urea, 2 M thiourea, 4% 3-[(3-cholamidopropyl) dimethylammonio]-1-propanesulfonate [CHAPS], and 40 mM Tris-HCl, pH 8.5). After homogenization by a grinder, protein was isolated from the mixture by Tris-saturated phenol. Crude protein products were precipitated from the phenol by using ammonium acetate/methanol. Protein pellet was washed with cold acetone and air dried. Next, the precipitant was solubilized in a SDS lysis buffer (SDSL3, 1% SDS with 1× cocktail protease, and 10 mM DTT), incubated at 56°C for 1 h, and treated by IAM (iodoacetamide) in the dark for 45 min. Protein products were precipitated by adding cold acetone and centrifuged at 25000×g for 20 min at 4°C. The pellet was dissolved into SDSL3. Protein concentration was measured by Bradford assay using bovine serum albumin (BSA) as a reference (Bradford, 1976).

### HPLC and Label-free LC-MS/MS

Protein digestion and high-performance liquid chromatography (HPLC) were performed according to previous methods with modifications (Yang et al., 2017). For each sample, 100 μg of protein was digested by 2.5 μg of trypsin (trypsin:protein = 1:40) for 12 h and desalted by using a Strata X C18 column. Mixed peptides (10 ug from each sample) were injected into a Shimadzu LC-20AB HPLC system with a Gemini high pH C18 RP column (4.6 × 250 mm, 5 μm, 110 Å) at a flowrate of 1 mL/min. Peptides were eluted with a binary mobile phase made by combining solvent A (5% ACN [pH 9.8]) and solvent B (95% ACN [pH 9.8]). The gradient of solvent B was set as follows: 5% solvent B for 10 min; 5%–35% solvent B for 40 min; 35%–95% solvent B for 1 min. The elution peak was monitored at a wavelength of 214 nm and component was collected every minute. Collected fractions were combined into ten fractions and dried by vacuum centrifugation.

LC-MS/MS (Liquid chromatography–mass spectrometry) was performed by using an UltiMate 3000 RSLCnano system coupled to a QExactive Obitrap HF-X mass spectrometer (Thermo Fisher Scientific). A nano-LC column (150 μm × 30 cm, 1.8 μm, 100 Å) was packed in-house as analytical column for peptide separation at a flowrate of 500 nL/min.

For DDA (data dependent acquisition) analysis, peptides were loaded onto a C18 trap column (300 μm × 50 mm, 5 μm, Thermo Scientific) with solvent A [2% ACN, 0.1% FA]. Peptides eluted from the trap column was loaded onto the analytical column at 500 nL/min in a gradient from 5%–25% solvent B [98% ACN, 0.1% FA] for 155 min; 25%–30% solvent B for 10 min; and 30%–80% solvent B for 5 min. The mass spectrometer was operated in a DDA mode. Survey full-scan MS spectra from 350–1500 m/z were acquired with a resolution of 120,000 at 400 m/z, and an automatic gain control (AGC) target value of 3e6. After the survey scans, MS/MS spectra were acquired in HCD (higher-energy collisional dissociation) mode at a resolution of 30,000 at 400 m/z, an AGC target value of 1e6, a maximum ion injection time (MIT) of 100 ms, an intensity threshold of 2e4, and dynamic exclusion duration of 30 s.

For DIA (data independent acquisition) analysis, the same nano-LC system and gradient was used as above DDA analysis. LC separated peptides were ionized by nanoESI and injected to the mass spectrometer with DIA detection mode. The MS parameters were set as below: full scan range 400–1250 m/z at resolution 120,000 with MIT 50 ms, and an AGC target value of 3e6. DIA isolation window was set to 17 m/z with loop count 50 and automatic MIT, scanned at the resolution of 30,000; stepped normalized collision energy (NCE): 22.5, 25, 27.5; an AGC target value: 1e6. The mass spectrometry proteomics data have been deposited in the ProteomeXchange Consortium *via* the PRIDE partner repository with the dataset identifier PXD036880.

### Proteomic data analysis

Raw MS/MS data from DDA mode were analyzed with MaxQuant version 1.5.3.30 (Tyanova et al., 2016). The *A. thaliana* proteome dataset (*Araport11_genes.201606.pep.fasta.gz*) which contains 40,785 entries were used as reference sequences. The false discovery rate (FDR) threshold of peptide-to-spectrum match (PSM) and proteins were set as 0.01. Minimal peptide length was set as 7. Cysteine carbamidomethyl was set as a fixed modification, while Methionine oxidation and N-termini acetylation were set as variable modifications. “Trypsin digestion” was chosen in the parameters setting while other searching parameters were kept as default. Identification results in MaxQuant were used for spectral library construction in Spectronaut. Raw MS/MS data from DIA mode were imported into Spectronaut for deconvolution and extraction (Pham and Jimenez, 2019). It used mProphet algorithm to perform analytical quality control and generate quantitative results. DEPs were identified by using MSstats package (Choi et al., 2014).

### Western blot analysis

Six-day-old seedlings of Col-0 and BES1-FLAG were transferred into new petri dishes with ½ MS solid medium containing 0 or 150 mM NaCl. At 0, 3, 6, 12 and 24 h of treatment, fresh samples were collected and frozen in liquid nitrogen. Then 100 mg of frozen tissues were taken for each sample and homogenized in liquid nitrogen, followed by boiling at 99°C for 10 min in the 2× sample buffer (0.125 M Tris-HCl [pH 6.8], 4% SDS, and 20% glycerol). After centrifuge at 14,000×g for 10 min, the supernatant was collected and separated by a 10% SDS-PAGE gel. Proteins in the gel were transferred to an Immobilon-P PVDF membrane by the eBlot L1 Fast Wet Transfer System (GeneScript, Nanjing, China). The PVDF membrane was sequentially incubated with a primary antibody anti-FLAG (FG4R, Invitrogen) and a secondary antibody Anti-Mouse IgG (H+L) (32430, ThermoScienifc). Blotting was detected by enhanced chemiluminescent horseradish peroxidase substrate (E412, Vazyme) on X-ray film (SUPER RX-N-C, FUJIFILM). The loading control was indicated by the level of Actin, which was detected by an anti-Actin antibody (AC004, Abclonal). The experiment was repeated three times.

### Reverse transcription and quantitative real-time PCR

Reverse transcription and qRT-PCR were conducted according to Luo et al. (2017). Total RNA was extracted from plant tissues by the EZNA Total RNA Kit II (Omega BIO-TEK). After quantification with NanoDrop 2000, one μg of total RNA of each sample was reverse transcribed with random hexamer primer by using the RevertAid First Strand cDNA Synthesis Kit (Thermo Scientific) according to its instructions. The cDNA products were diluted 10 folds with sterilized milli-Q water and used as templates. Each reaction mixture (20 μL) contains 2 μL of cDNA templates, 4 μL of forward primer (1 μM), 4 μL of reverse primer (1 μM), and 10 μL of 2X premixed reagent (SYBR Premix Ex Taq II, Tli RNase H Plus, TaKaRa). A two-step PCR protocol was used in CFX96 Touch™ Real Time PCR Detection System (BioRad) by using the following procedures: 95°C for 3 min, followed by 39 cycles of 95°C for 10 s and 60°C for 30 s. A housekeeping gene *UBQ10* was used as an internal standard. Primer sequences for qRT-PCR were listed in **Supplementary Table S3**. Three independent biological repeats were performed for each sample. Relative expression of each interested gene was calculated using 2^(−ΔΔCt) method (Livak and Schmittgen, 2001). ANOVA and Tukey’s HSD were used for evaluation of change significance.

### Luciferase luminescence assays

Total transcriptional activation analyses of BES1 (cloned from Col-0) and BES1-D (cloned from *bes1-D*) were carried out by using the dual-luciferase reporter (DLR) assay system with slight modifications (Feng et al., 2019; Wang et al., 2019). The CDS of *BES1* and *BES1-D* were respectively cloned into *pGreenII-62-SK* vector as the effector constructs, and the 2-kb upstream sequences of *NHX2*, *CAX3*, *SLAH3*, and *HKT1* were cloned into *pGreenII-0800-LUC* vector as the reporter constructs. Primer sequences used for making the effector and reporter constructs were listed in **Supplementary Table S4**. The successfully constructed plasmids were enriched through a plasmid extraction kit (DNA-spin™, Intron Biotechnology). Protoplasts from four-week-old seedlings grown under short photoperiod (10 h light/14 h dark at 20°C) were prepared using enzymatic hydrolysate (1.5% cellulose R10 and 0.4% Macerozyme R-10). Subsequently, the effector and reporter construct plasmids were mixed at a ratio of 4:1 in a 2-mL centrifuge tube and transferred into 100 μL of *Arabidopsis* protoplasts (20,000 cells) by PEG-Calcium mediated transfection. After transformation, the protoplasts were incubated at 25°C in the dark for 12–16 h, and harvested by centrifugation at 100×g for 2 min. To rupture these protoplasts, 100 μL of lysis buffer was added and mixed vigorously by vortexing for 2 s. Then, they were placed on ice for 5 min and centrifuged at 1000×g for 2 min. Finally, 10 μL of lysate was mixed with 40 μL of luciferase assay buffer (Promega). Firefly luciferase activity (LUC) and Renilla luciferase activity (REN) were measured by using a luminometer (BioTek Synergy H1 Multi-Mode Microplate Reader). Final transcriptional activities were calculated based on the ratio of LUC to REN. The experiments were repeated three times. Student’s *t* test was used for evaluating significance of change.

### Electrophoresis mobility shift assay

For recombinant protein production, a truncated *BES1*(without nuclear localization signal) was first cloned into *pMal-C2X* vector in frame with a C-terminal MBP tag. MBP-BES1 fusion protein was purified with amylose agarose beads from *Escherichia coli* BL21 cell line. Then, the presence of E-box (CAnnTG) and BRRE (CGTG[T/C]G) motifs was examined in the 2-kb promoter regions of putative target genes. Oligo probes were synthesized and labeled with biotin using Biotin 3’ End DNA Labeling Kit (Thermo Scientific, Cat. #89817). The labeled single strand probes were annealed into double strands and EMSA was performed using LightShift^®^ Chemiluminescent EMSA Kit (Thermo Scientific, Cat. #20148). Briefly, 2 μg of purified MBP-BES1 was incubated with biotin-labeled promoter probes at 25°C for 30 min according to the manufacturer’s instructions, and 500, 1000 and 2000-fold of cold probes (without 3’-biotin labeling) and E-box or BRRE-mutated mutant cold probes were used for competition assay with the biotin-labeled probes. The results were detected by Chemiluminescent Nucleic Acid Detection Module Kit (Thermo Scientific, Cat. #89880). All the DNA probes synthesized for EMSA were listed in **Supplementary Table S5**.

## Results

### The *bes1-D* mutant showed higher salt tolerance than the wild-type plants

**Figure 1:**
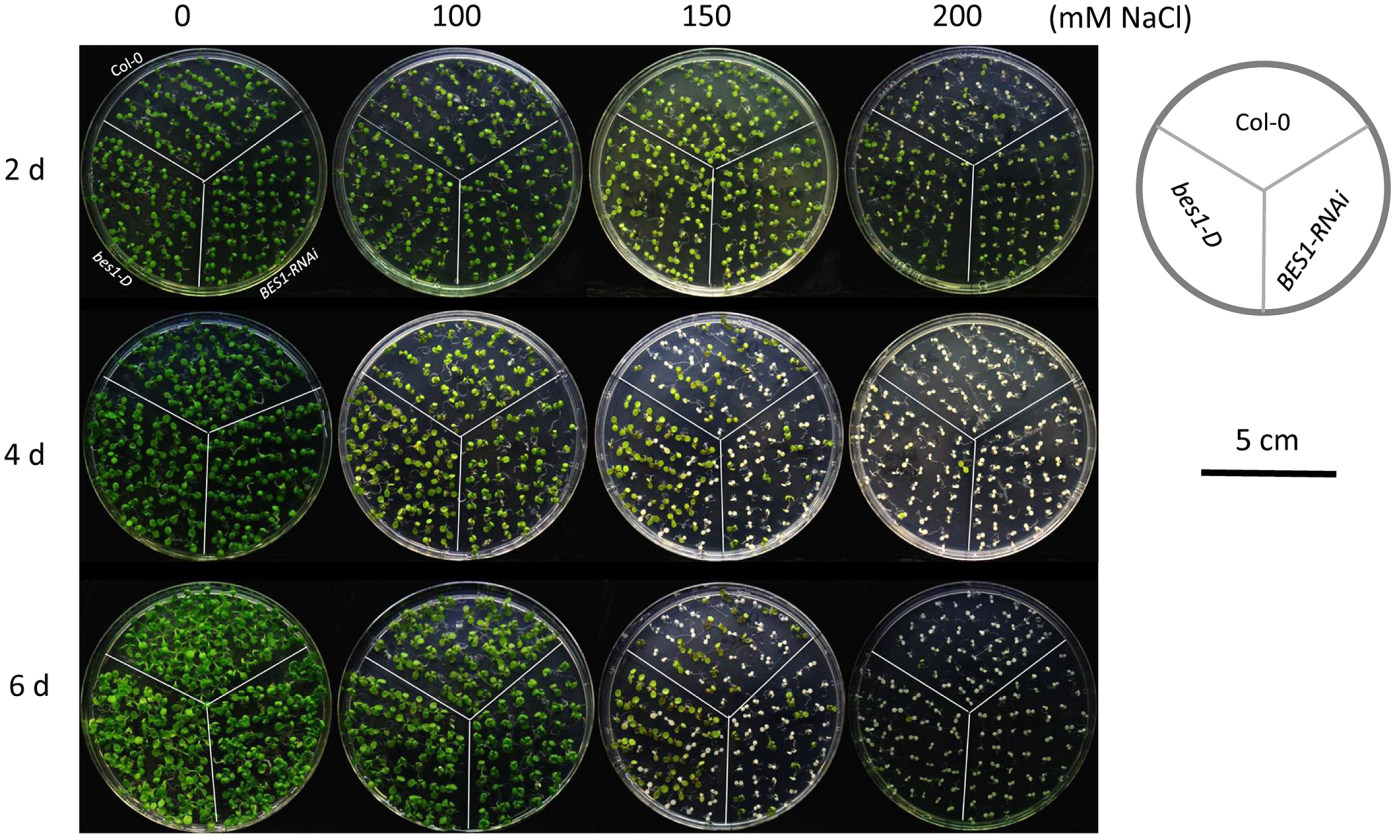
Phenotypic analysis of *bes1-D* and *BES1-RNAi* under salt stress. Col-0, a wild type; *bes1-D*, a gain-of-function mutant for BES1; *BES1-RNAi*, a loss-of-function mutant for BES1. Growth media with different concentrations of NaCl (0, 100, 150 and 200 mM) were used for the treatment. Photos were taken at the 2nd, 4th, and 6th day of the treatment.

**Figure 2:**
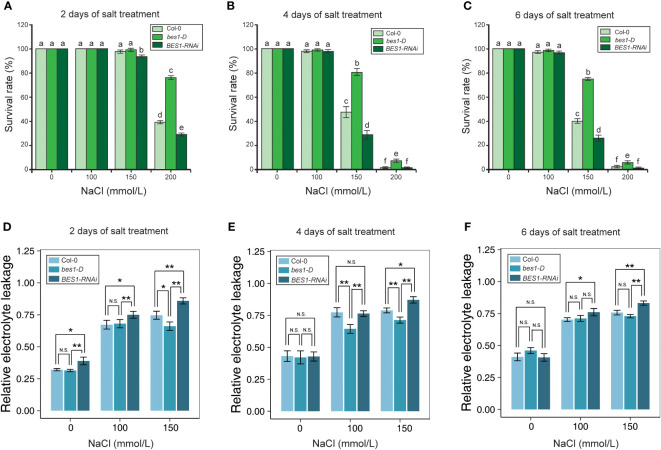
Physiological analysis of Col-0, *bes1-D*, and *BES1-RNAi* under salt stress. **(A–C)** Survival rates of *Arabidopsis* at the 2nd **(A)**, 4th **(B)** and 6th **(C)** day of salt treatment. Five biological replicates were performed. For each replicate, 50 seedlings were planted. Values are means ( ± SE) of five replicates. Statistically significant differences (*P* < 0.05) were calculated by using one-way ANOVA with LSD *post-hoc* analysis. **(D–F)** Relative electrolyte leakage of *Arabidopsis* in 2nd **(D)**, 4th **(E)** and 6th **(F)** day of salt treatment. Values are means ( ± SE) of nine replicates (*t* test, * *P* < 0.05, ** *P* < 0.01, N.S, not significant.) Different lowercase letters above bars denote significant differences of change (P < 0.05).

To assess the responses of Col-0, *bes1-D* and *BES1-RNAi* under salt stress, six-day-old seedlings were transferred to growth media with different concentrations of NaCl (0, 100, 150, and 200 mM). All the three plant materials performed similarly under 100 mM NaCl treatment, with an average survival rate > 97.17% after six days’ treatment. Significant differences in survival rates of three plant materials were observed after 4 days of 150 mM NaCl treatment. *bes1-D* showed a much higher survival rate (76.88%) than Col-0 (47.61%) and *BES1-RNAi* (28.66%) at the 4th day of treatment. Similar phenotypic changes were also found at the 6th day of treatment with 150 mM NaCl. Growth of all the plant materials was severely affected by 200 mM NaCl treatment even at the early stage (2 d), but the *bes1-D* mutant still showed a much higher survival rate (75.71%) compared to the wild-type Col-0 (38.9%) and the *BES1-RNAi* line (28.77%). At the 4th day of treatment with 200 mM NaCl, the survival rates of all three materials decreased dramatically, but *bes1-D* remained the highest (6.57%), compared with that of Col-0 (1.94%) and *BES1-RNAi* (1.05%) (**Figures 1**, **2A–C**). Electrical conductivity analysis confirmed that *bes1-D* had lower relative electrolyte leakage than Col-0 and *BES1-RNAi* under salt stress, particularly under 150 mM NaCl treatment (**Figures 2D–F**). These results demonstrate that BES1 is involved in plant responses to salt stress and gain of BES1 function could enhance salt tolerance in *Arabidopsis*, whereas knocking down of BES1 increases plant sensitivity to salt treatment.

### Transcriptome sequencing revealed gene clusters specifically regulated by BES1 under salt stress

**Figure 3:**
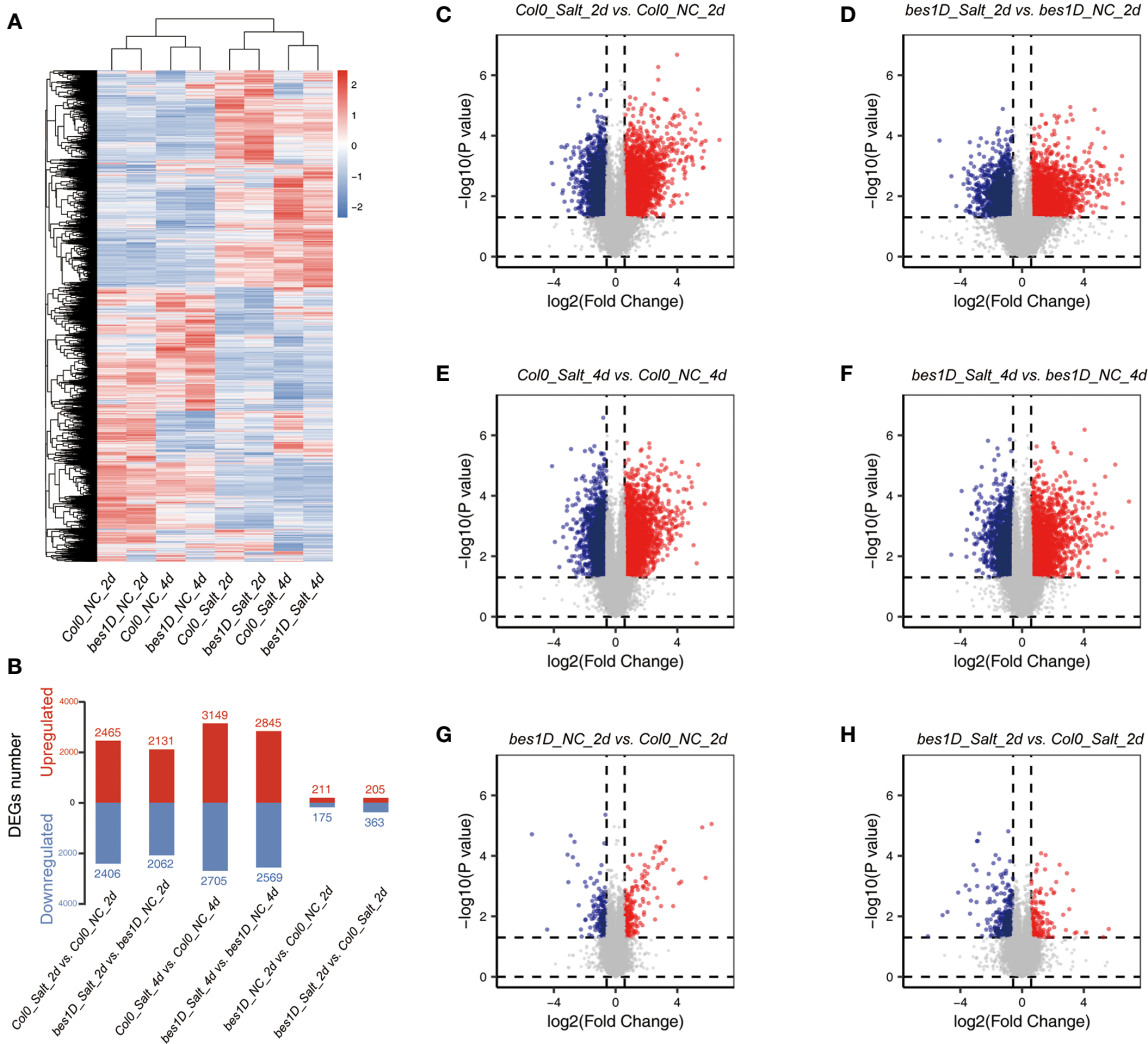
Transcriptomic analyses in *bes1-D* and Col-0 under salt stress. **(A)** Heatmap of genes in eight experimental groups. A total of 13,528 genes with average FPKM > 5 were used for this analysis. FPKM values were transformed by Z-score = (FPKM − mean)/σ. Hierarchical cluster based on Euclidean Distance was used for the clustering analysis. NC, normal condition, or negative control; Salt, 150 mM NaCl. **(B)** DEGs numbers in different comparisons. Fold change (absolute value) > 1.5 and P value < 0.05 were regarded as DEGs. Red, upregulated genes; blue, downregulated genes. **(C–H)** Volcano plot of all expressed genes in different comparisons. Red, significantly upregulated genes; blue, significantly downregulated genes.

On the basis of the phenotypic data, we used 150 mM NaCl for salt treatment to investigate global gene expression changes. Seedlings of *bes1-D* and Col-0 were collected after 2 days and 4 days of the treatment (**Supplementary Table S1**). In total, 24 plant samples were used for RNA isolation, library construction, and transcriptome sequencing. In average, 28.84 M of read pairs were produced from each sample, and 97.31% of these read pairs can be aligned to the *Arabidopsis* genome (**Supplementary Table S2**). Gene expression profiling showed that 29,669 genes were expressed in at least one sample. Correlation coefficient between each pair of samples in three biological replicates were not less than 0.91, indicating good consistency of independent biological replicates (**Supplementary Figure S1**). Hierarchical clustering based on Euclidean Distance of the eight samples showed that salt-treated and untreated samples were obviously separated into two clusters. Furthermore, genes in the 2-day and 4-day samples were separated into sub-clusters, which indicated more detailed global gene expression changes from 2-day to 4-day of the treatment (**Figure 3A**). DEGs were identified between treated samples and controls. Under the salt stress, around 60% DEGs were shared by *bes1-D* (*bes1D_Salt_2d* vs. *bes1D_NC_2d*) and Col-0 (*Col0_Salt_2d* vs. *Col0_NC_2d*), suggesting that thousands of genes were specifically regulated in *bes1-D*. Even for the same materials, only ~50% of the DEGs were identical at different time points of the treatment, indicating significant gene expression changes in response to salt stress (**Supplementary Figure S2**). It was also found that the number of DEGs in 4-day samples was larger than that of 2-day samples (**Figures 3B–F**). But DEGs at 2-day changed more dramatically than 4-day, as evidenced by a more dispersed volcano plot of 2-day samples (**Figures 3C–D**) than 4-day samples (**Figures 3E–F**). For example, a well-known stress-induced gene *WRKY25* (Zheng et al., 2007) was upregulated by nearly 13 folds in 2-day samples, but only 2.62 folds at 4-day samples. Similar expression pattern was also found for another stress-induced gene *WRKY48* (Xing et al., 2008) (**Supplementary Figure S3**). We also examined the expression of two BR biosynthetic genes *CPD* and *DWF4*, which are repressed by BR signaling through a negative feedback regulation (He et al., 2005). Indeed, both *CPD* and *DWF4* had lower expression levels in *bes1-D* compared to Col-0. Besides, the expression of *CPD* was downregulated to around two folds in salt-treated samples compared to control samples. Surprisingly, the expression of *DWF4* only slightly downregulated in response to salt stress (**Supplementary Figure S4**).

**Figure 4:**
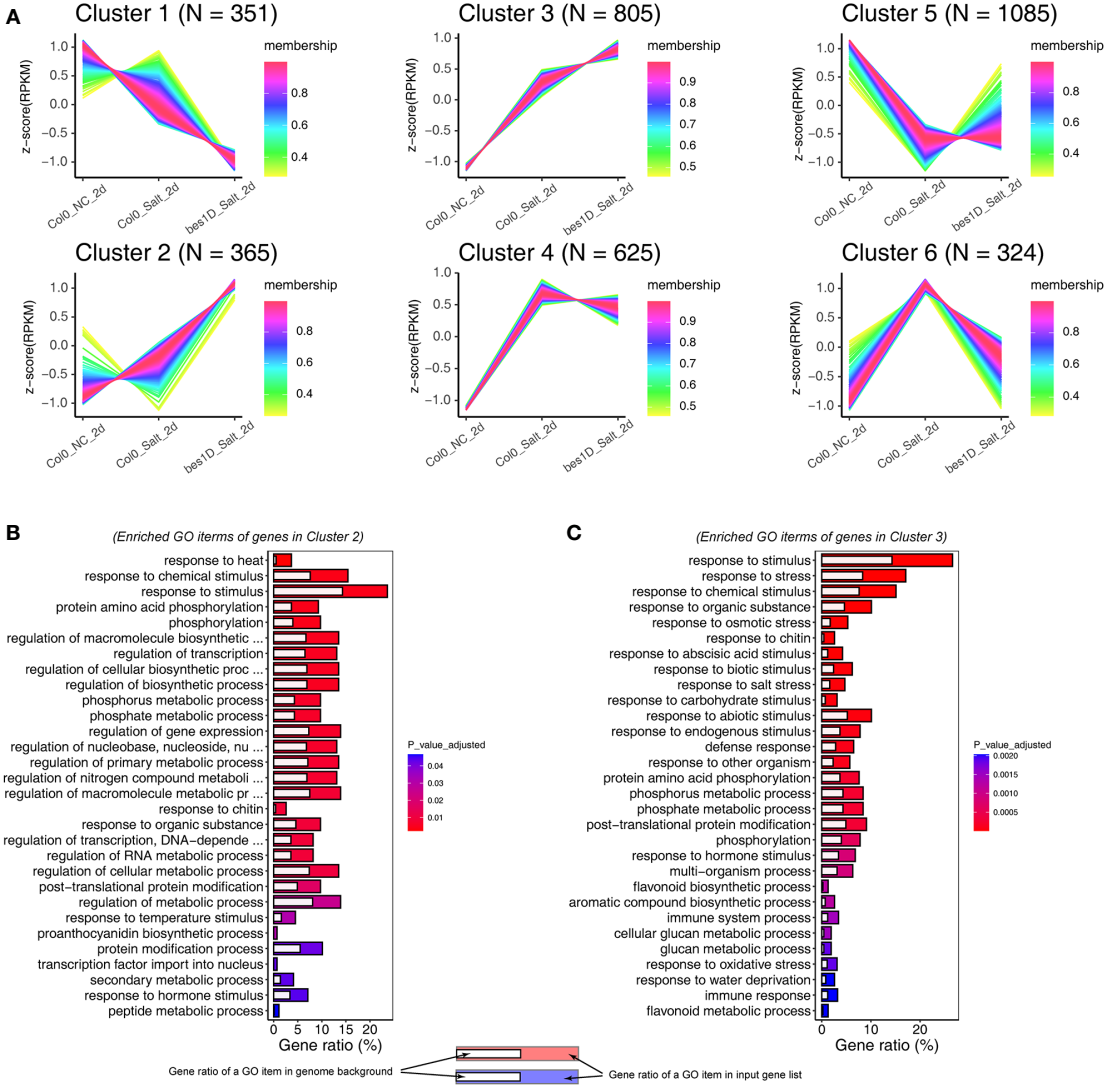
Time Series Clustering analyses of genes in *bes1-D* and Col-0 under salt stress. **(A)** Fuzzy c-means algorithm was used for clustering. Gene expression was normalized by Z-score transformation. **(B)** Gene Ontology analysis for 365 genes in cluster 2. **(C)** Gene Ontology analysis for 805 genes in cluster 3. Only top 30 Biological Process items were showed in **(B, C)** due to the space limitation.

Given the distinct gene expression changes at 2-day of the salt treatment, we used *Col0_NC_2d* (Col-0 under normal conditions at 2-day), *Col0_Salt_2d* (Col-0 under treatment of 150 mM NaCl for 2 days), and *bes1D_Salt_2d* (*bes1-D* under treatment of 150 mM NaCl for 2 days) to perform a time series clustering analysis through a Fuzzy C-means clustering algorithm. From this analysis, DEGs were clustered into six clusters (**Figure 4A**; **Supplementary Figure S5**; **Supplementary Table S5**). In Cluster 1, 351 genes were only slightly downregulated in *Col0_Salt_2d*, but they were greatly downregulated in *bes1D_Salt_2d*. These genes were significantly enriched in biological processes related to plant growth and development, such as shoot system development, cotyledon development, and organ formation (**Supplementary Figure S6A**). Notably, the most significant GO item in Cluster 1 was BR biosynthesis process which contains *CPD*, *DWF4, BR6OX2, CYP90D1*, and *ST4A*. Besides, a key gene for ROS generation, *RBOHB*, was also detected in Cluster 1, which suggested reduced ROS generation in *bes1-D*. In Cluster 2, 365 genes were clearly upregulated in *bes1D_Salt_2d* but barely changed in *Col0_Salt_2d*. Functional annotation of these genes demonstrated that many of them are transcription factors related to stress responses or gene regulation (**Figure 4B**). Cluster 3 contains 805 genes which were slightly upregulated in *Col0_Salt_2d* but significantly upregulated in *bes1D_Salt_2d*, suggesting that the expression of these genes was promoted by BES1 to enhance plant salt tolerance. Functional annotation of these 805 genes implied that they were enriched in processes such as response to multiple stresses (e.g., salt stress, osmotic stress, oxidative stress, and defense response), and flavonoid biosynthesis (**Figure 4C**). Notably, two stress-responsive genes *WRKY25* and *WRKY48* mentioned above were also found in Cluster 3. In addition, a series of key genes involved in anthocyanin biosynthesis were also among the upregulated genes in Cluster 2 (e.g., *PAL2*, *4CL3*) and Cluster 3 (e.g., *DFR*, *TT4*, *TT7*, *TT8*, *UF3GT*), indicating BES1 may target them to enhance anthocyanins biosynthesis and cope with the stress by acting as ROS scavengers, photoprotectants, and/or stress signals. Functional enrichment analyses were also performed for Cluster 4**–**6 (**Supplementary Figures S6B–D**). In Cluster 4, 625 genes were upregulated under salt stress, but they did not show much more changes between *Col0_Salt_2d* and *bes1D_Salt_2d* samples. We found one biological process “response to ABA stimulus” was enriched in Cluster 4, suggesting that ABA signaling is also an important mechanism of salt tolerance in *bes1-D*. Cluster 5 contained the largest number of genes (1,085) among the six clusters. However, all adjusted *P* values were > 0.05 for GO biological processes generated by enrichment analysis of 1,085 genes in the Cluster 5 (**Supplementary Figure S6C**). In Cluster 6, 324 genes were significantly upregulated in *Col0_Salt_2d*, but with little changes in *bed1D_Salt_2d*. A large portion of Cluster 6 genes were enriched in processes such as response to stimulus and response to stress (**Supplementary Figure S6D**). Interestingly, *RD26*, a gene that is transcriptionally repressed by BES1 (Ye et al., 2017) was observed in Cluster 6.

### qRT-PCR validation of selected DEGs

**Figure 5:**
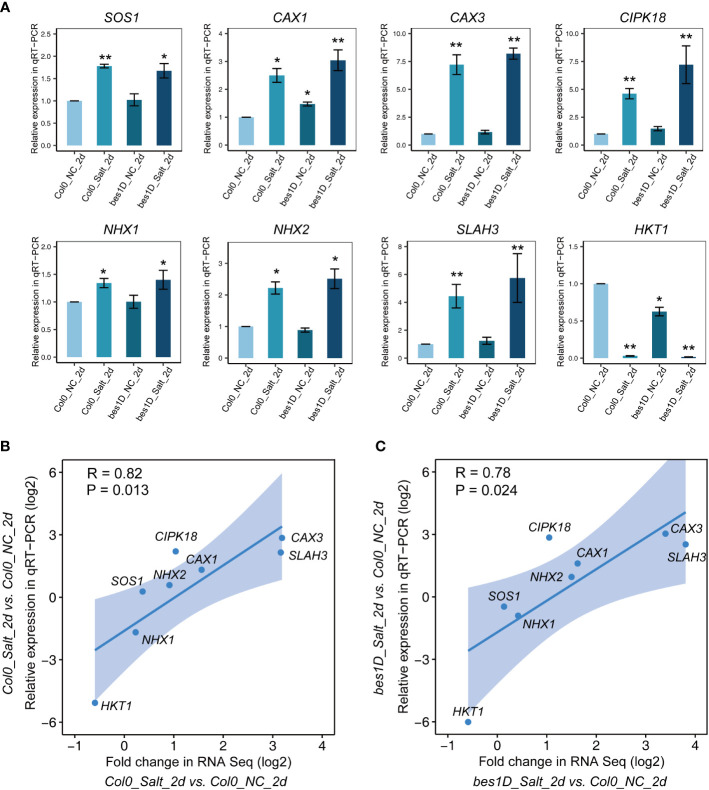
Validation of selected genes by qRT-PCR and the correlation coefficient analysis of qRT-PCR and RNA seq. **(A)** qRT-PCR results of *SOS1*, *CAX1*, *CAX3*, *CAX7*, *NHX1*, *NHX2*, *SLAH3*, and *HKT1* in Col-0 and *bes1-D* under salt stress. NC, normal conditions; Salt, 150 mM of NaCl. The sample *Col-0_NC_2d* was used as control for other experimental samples. A housekeeping gene *UBQ10* (AT4G05320) was adopted as an internal reference for normalization of gene expression values. Relative expression values were calculated by using 2^(−ΔΔCt) method. Error bar means SE value of three biological repeats for each gene. Stars above error bars denote significant differences compared to *Col-0_NC_2d* (* *P* < 0.05, ** *P* < 0.01, ANOVA and Tukey’s HSD). **(B, C)** Evaluation of the correlation coefficient between fold changes in RNA Seq and relative expression in qRT-PCR of selected genes. Pearson correlation coefficient between the two groups and its significance (*P* value) were calculated by using *ggpubr* package in RStudio.

It has been well established that excess Na^+^ caused by salt stress triggers a cytoplasmic Ca^2+^ signaling, which will change the activities of ion transporters for Na^+^, K^+^, and H^+^, and thus reduce Na^+^ accumulation in cytoplasm and maintain the ion homeostasis in the cell (Zhu, 2002). To correlate the gene expression changes in transcriptome with ion transporters, a total of eight genes involved in the SOS signaling pathways were selected to validate their expression changes by qRT-PCR (**Figure 5A**). *SOS1*, encoding a plasma membrane Na^+^/H^+^ antiporter, is stably expressed in all the transcriptome samples with an average FPKM of 5.1 ± 1.1 (mean ± SD), with little expression changes between *bes1-D* and the wild-type, under either normal or salt stress conditions. However, significant expression changes were observed for the cation exchanger genes *CAX1* and *CAX3*, which encode vacuolar ion transporters contributing to cellular ion homeostasis. In transcriptome, *CAX1* was enriched in Cluster 3 and *CAX3* was in Cluster 4. Similar expression patterns were observed in qRT-PCR and transcriptome data for these two genes. CIPK18, a CBL-interacting protein kinase responsible for activating Na^+^/H^+^ antiporters, was also found upregulated in Cluster 3 and its expression was validated by qRT-PCR with high significance of change. The *NHX1* gene (for transporting of Na^+^ or K^+^ into the vacuoles) was stably expressed in all transcriptome samples with an average FPKM of 28.6 ± 4.3 (mean ± SD), and this was confirmed by qRT-PCR analysis. *NHX2* showed slight upregulation under salt stress in both transcriptome and qRT-PCR analysis, but the upregulation is statistically insignificant in the two datasets. A gene encoding the slow-type (S-type) anion channel protein SLAH3 was upregulated in Cluster 3 of the transcriptome data, and it was clearly induced by salt stress in qRT-PCR data. HKT1 controls Na^+^ entry into cytoplasm and its expression was significantly repressed under salt stress in transcriptome and this was validated by qRT-PCR analysis. We also checked the expression changes of *BES1* mRNA which was down-regulated by 2 days’ NaCl treatment (150 mM) in the WT but not in *bes1-D*, indicating that *BES1* expression was less affected in *bes1-D*.(**Supplementary Figure S7**). Overall, the Pearson correlation coefficient between RNA seq and qRT-PCR data of *Col0_Salt_2d* vs. *Col0_NC_2d* is 0.82 (*P* value = 0.013, **Figure 5B**), while that of another comparison pair (*bes1D_Salt_2d* vs. *Col0_NC_2d*) is 0.78 (*P* value = 0.024, **Figure 5C**), suggesting good consistency of the two datasets.

### Salt-induced proteomic responses and comparative analysis with the transcriptome data

**Figure 6:**
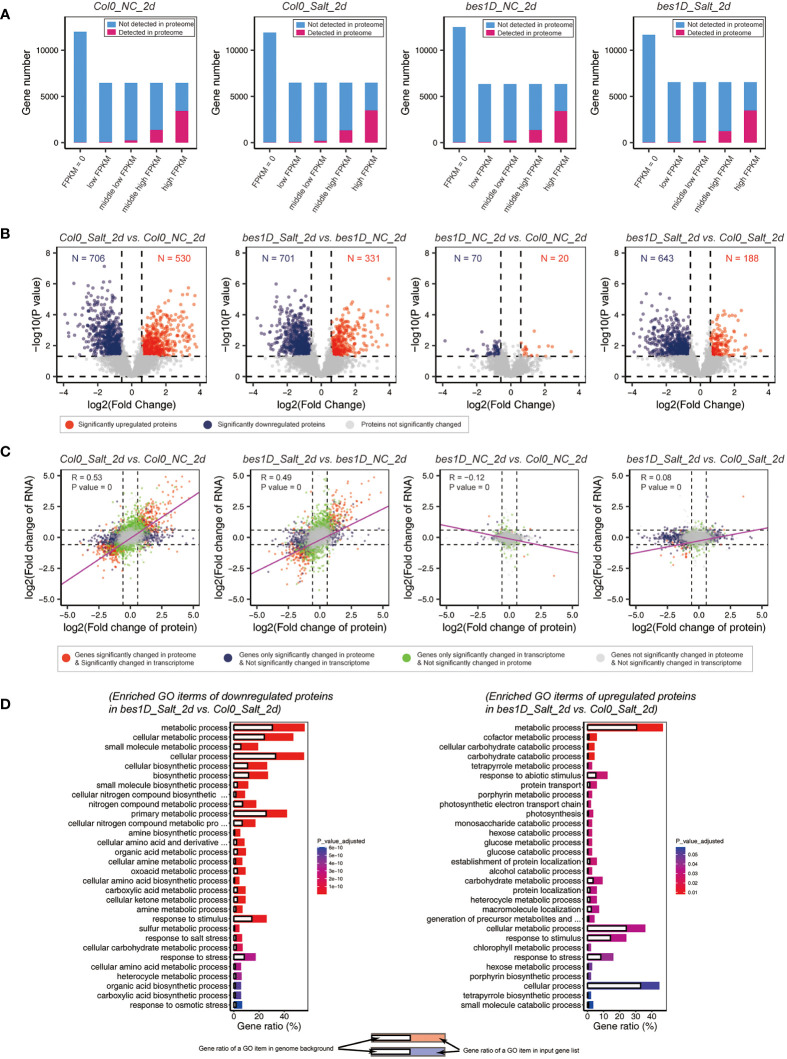
Proteomic analyses in *bes1-D* and Col-0 under salt stress. **(A)** Overlapping analysis of proteomic and transcriptomic data. All gene entries of *A thaliana* were classified into five groups based on their RNA-level FPKM values. “FPKM = 0” are genes that no reads were found in transcriptome. Expressed genes (FPKM > 0) were sorted by FPKM values and divided into four quarters: low FPKM, middle-low FPKM, middle-high FPKM, and high FPKM. Proteins identified in LC-MS/MS data were mapped to these five categories. NC, normal conditions; Salt, 150 mM NaCl; 2d, 2 days of the treatment. **(B)** Volcano plot of all detected proteins in different comparisons. Fold change (absolute value) > 1.5 and *P* value < 0.05 were regarded as DEPs. Red, significantly upregulated genes; blue, significantly downregulated genes. **(C)** Correlation coefficient analysis of proteomic data and transcriptomic data. For genes both detected by RNA seq and LC-MS/MS, their fold changes were collected and transformed *via* log2, respectively. Pearson correlation coefficient between the two groups and its significance (*P* value) were calculated by using *rcorr* package in RStudio. Linear regression (purple lines) was performed by using fitting linear models (*lm* function). **(D)** Gene Ontology enrichment analysis for downregulated and upregulated DEPs in *bes1-D_Salt_2d* vs. *Col-0_Salt_2d*. Only top 30 Biological Process items were showed for each group due to the space limitation.

Seedlings of *bes1-D* and Col-0 collected at the 2nd day of treatment 150 mM NaCl were also used for quantitation of proteins by label-free LC-MS/MS. For the 37,866 gene entries in *Arabidopsis* genome (TAIR10), an average of 5,062 ± 51 (mean ± SD) coded proteins were detected in the proteomic samples. As a comparison, a total of 25,841 ± 349 genes were detected in transcriptome samples. As a much larger number of genes were detected by transcriptome sequencing than by proteome profiling, we performed an overlapping analysis of proteins based on their FPKM values for RNA levels. For each sample, all *Arabidopsis* genes were classified into five categories based on their expression values (FPKM) in transcriptome. “FPKM = 0” means that no reads were found for genes in transcriptome data. Expressed genes (FPKM > 0) were ascendingly sorted by FPKM values and divided into four quarters: low, middle-low, middle-high, and high FPKMs. Proteins identified in proteomic data were mapped to these five categories. In all the four samples analyzed, more than 93% of proteins belong to “middle-high” and “high FPKM” groups. More than 67% of the LC-MS/MS detected proteins belong to the “high FPKM” group. Even for all the genes in “high FPKM” group, around 47% of them were not detected by proteomics (**Figure 6A**). Next, differentially expressed proteins (DEPs) were identified by using MSstats. In Col-0, 530 proteins were upregulated, and 706 proteins were downregulated under salt stress. In *bes1-D*, similar numbers of DEPs were identified. A total of 90 DEPs (20 upregulated and 70 downregulated) were found in *bes1-D* over Col-0 under normal conditions. The small number of DEPs might reflect that these two materials have similar proteomes during their young seedling stage. However, a larger number of differentially expressed proteins (188 upregulated and 643 downregulated) were found in *bes1-D* over Col-0 under salt stress (**Figure 6B**), suggesting that *bes1-D* might specifically regulate some target genes involved in salt stress response. We evaluated the association of proteome and transcriptome by Pearson correlation coefficient. By comparing the *Col0_Salt_2d* vs. *Col0_NC_2d* samples and a correlation coefficient of 0.53 was observed between transcriptome and proteome data. Another comparison of *bes1D_Salt_2d vs. bes1D_NC_2d* revealed a coefficient of 0.49 between transcriptome and proteome. Weak correlation was found for comparisons of materials under the same conditions regardless of plant genotypes (i.e., *bes1D_NC_2d* vs. *Col0_NC_2d*; *bes1D_Salt_2d* vs. *Col0_Salt_2d*) (**Figure 6C**). Gene Ontology enrichment analysis of DEPs in *bes1D_Salt_2d* vs. *Col0_Salt_2d* showed that proteins related to metabolic process and response to stimulus, response to salt stress were the mainly enriched items (**Figure 6D**).

### Network construction identified key modules affected in *bes1-D* under salt stress

**Figure 7:**
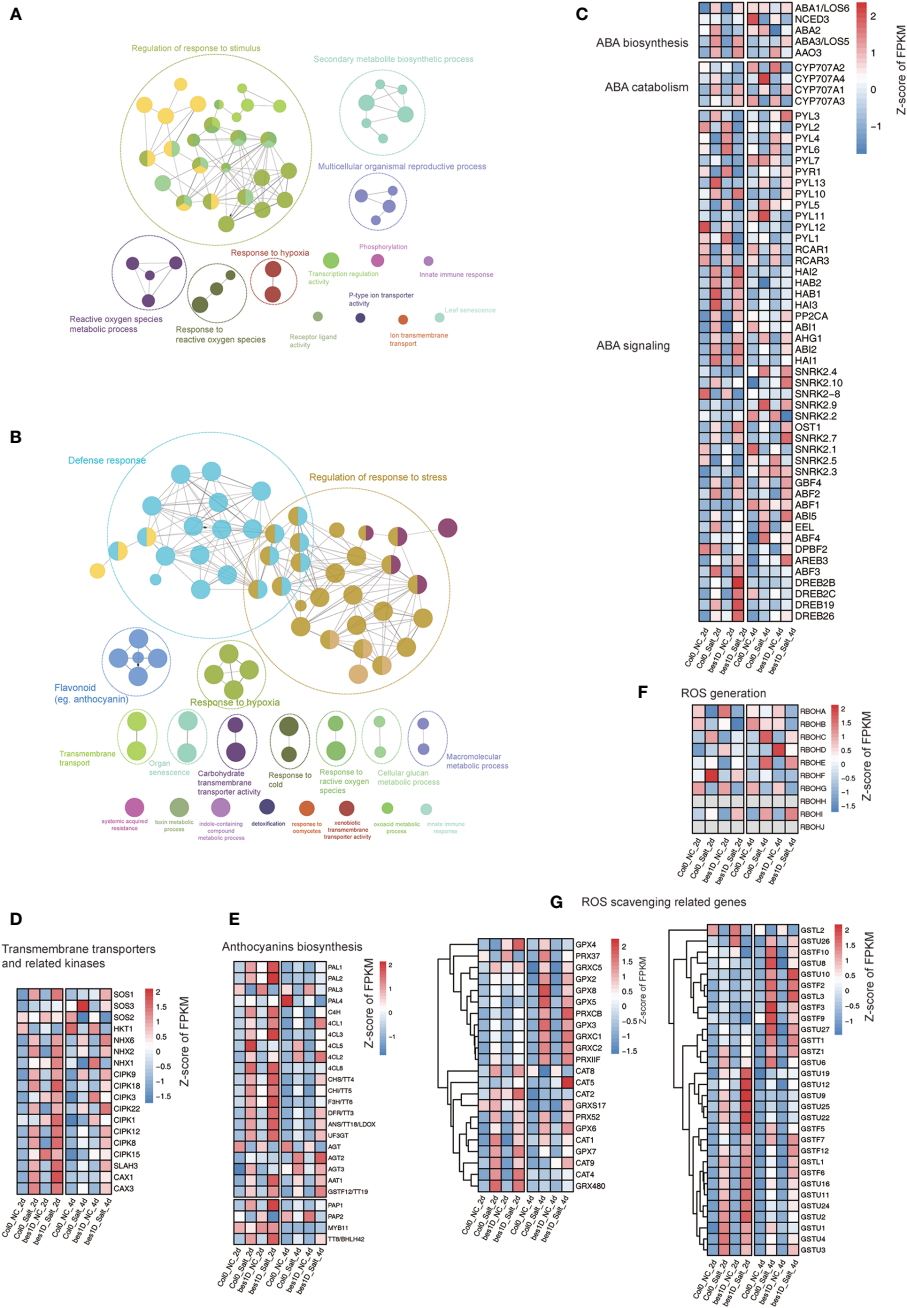
Network and pathways analyses of key modules affected in *bes1-D*. Gene Ontology enrichment network of genes in Cluster 2 **(A)** and Cluster 3 **(B)**. Expression profiling of genes evolved in ABA biosynthesis, catabolism, and signaling **(C)**; Na^+^/K^+^ ion transportation **(D)**; Anthocyanins biosynthesis **(E)**; ROS generation **(F)**; and ROS scavenging **(G)**.

The clustering and functional enrichment analyses have revealed that a series of biological processes were promoted in *bes1-D* under salt stress (**Figures 4B, C**). Considering the high redundancy in the enriched GO items, we performed a GO enrichment network analysis based on transcriptome data to produce a more concise scenario. In the network of Cluster 2, “regulation of response to stimulus” is the predominant module (22 nodes out of 52 total nodes). Other important modules include secondary metabolic biosynthetic processes, cell surface receptor signaling pathways, responses to ROS, etc. (**Figure 7A**). In the network of Cluster 3, “defense response” and “regulation of the response to stress” are two main modules in the network. The rest are flavonoid biosynthesis, response to hypoxia, transmembrane transport, responsive to reactive oxygen species, organ senescence, and carbohydrate transmembrane transporter activity, etc. (**Figure 7B**). ABA signaling pathway is one of key pathways in response to salt stress in plants. A heatmap of genes involved in ABA biosynthesis, catabolism, and signaling demonstrated that ABA signaling pathway was activated under salt stress, and some *DREB* genes (*DREB2B* in Cluster 2; *DREB2C*, *DREB19*, and *DREB26* in Cluster 3) were found to be upregulated in *bes1-D* (**Figure 7C**), suggesting that BES1 may enhance the expression of ABA responsive genes under salt stress. “Transmembrane transport” was a key module in the network. For example, transporters-related genes such as *SOS* family genes, *NHX* family genes, and *SLAH3* showed enhanced expression in *bes1D_Salt_2d* compared to *Col0_Salt_2d*. The kinase CIPK family that is responsible for activating transmembrane transporters was also activated in *bes1D_Salt_2d* (**Figure 7D**). Anthocyanin biosynthesis belongs to the “Flavonoid biosynthesis” module, and expression heatmap showed that many of the anthocyanin biosynthesis genes were upregulated in *bes1D_Salt_2d* (**Figure 7E**). ROS acts as a second messenger to regulate various stress responses (Pitzschke et al., 2006). To balance the ROS accumulation, plants have evolved the effective enzymatic antioxidant defense system including catalase (CAT), peroxidase (POD), superoxide dismutase (SOD), and polyphenol oxidase (PPO) enzymes to scavenge ROS and protect plant cells from oxidative damage. We characterized the expression pattern of *RBOH* genes related to ROS generation and found that the expression of *RBOHB, RBOHC*, and *RBOHF* was inhibited in *bes1-D*_*Salt_2d* (**Figure 7F**). Consistently, the expression of some genes of the glutathione S-transferases (GSTs) family (e.g., *GSTU19*, *GSTU12*, *GSTU9*, *GSTU25*, *GSTU22*) were also enhanced in *bes1D_Salt_2d* (**Figure 7G**).

### BES1 directly binds and regulates the expression of some salt-responsive genes

**Figure 8:**
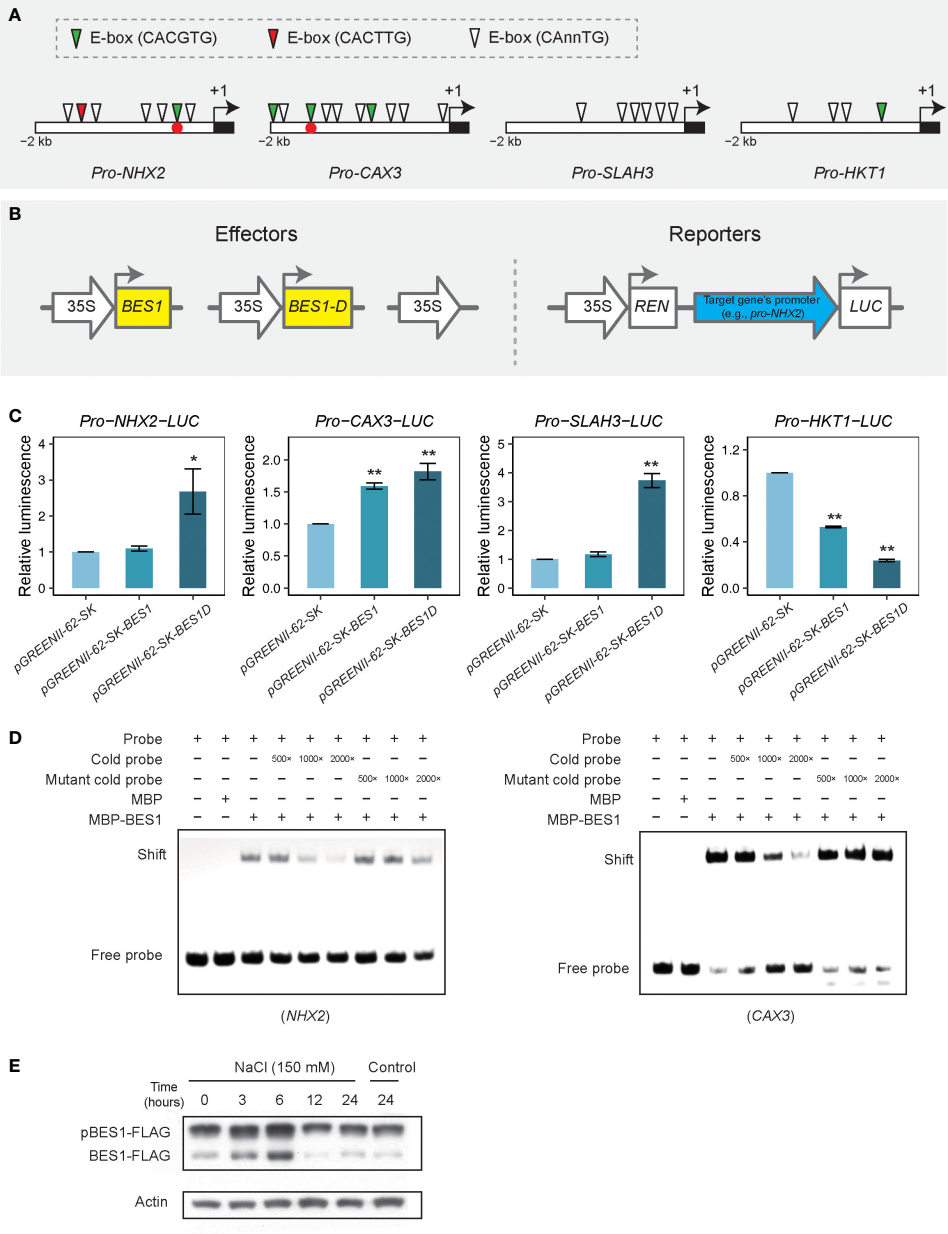
Transient expression assay for transcriptional activation or repression activity of BES1 on target genes by using luciferase luminescence and EMSA. **(A)** Motif analysis in promoters of putative target genes. Empty boxes represent upstream 2-kb of open reading frame (ORF) region. Black block means start codon and arrows are translational direction. **(B)** Transient expression assay and construct structures. Thick arrows denote promoters while rectangles are coding sequence (CDS) regions. Upstream 2-kb fragments of target genes were inserted to constructs for driving *LUC* expression. BES1-D (or BES1D) means it was cloned from the *bes1-D* mutant. **(C)** Transient assay for target genes regulated by BES1 or BES1-D. Values are means ( ± SE) of at least three replicates. Asterisks are significant differences between BES1/BES1-D and a positive control *pGREENII-35S_pro_-LUC* (*t* test, * *P* < 0.05, ** *P* < 0.01). **(D)** EMSA analysis of BES1 binding to the promoter regions of *NHX2* and *CAX3* genes. Cold probes (without biotin labeling) or mutated cold probes were used to compete with the labelled probes for binding with BES1. Positions of these two binding motifs were labeled as red dots in panel **(A)**. **(E)** Western blot analysis of BES1-FLAG protein level under salt treatment (150 mM of NaCl) at different time points. A BES1-FLAG overexpressing line was used for the salt treatment. pBES1-FLAG and BES1-FLAG denote the phosphorylated and dephosphorylated forms of BES1, respectively. Actin was used as a loading control.

As the time series clustering analysis and functional enrichment network analysis demonstrated that many genes were specifically changed in *bes1-D* under salt stress, we hypothesized that some of these genes may be directly regulated by BES1 though binding to their promoters. We selected four genes (*NHX2*, *CAX3*, *SLAH3*, and *HKT1*) to check whether there are any BES1-binding motifs BRRE (CGTG[T/C]G) and/or E-box (CAnnTG) in their promoters. Four to ten E-box motifs but no BRRE motifs were found in the upstream 2-kb fragments of these four genes (**Figure 8A**). Transient transactivation assays in *Arabidopsis* protoplasts were used to examine whether BES1 (cloned from Col-0) or BES1-D (cloned from *bes1-D*) can activate/repress the transcription of these salt-responsive genes (**Figure 8B**). Three effector vectors (BES1, BES1-D, and vector control) were co-transformed with a reporter vector harboring the promoters of the selected genes, respectively. The results showed that BES1-D induced significant higher luciferase expression driven by the *NHX2* and *SLAH3* promoters compared to that driven by the vector control, while BES1 did not induce significantly higher luciferase activity relative to the control. However, both BES1-D and BES1 caused significant luciferase expression driven by the *CAX3* promoter than the control. In the co-transformation of *pro-HKT1-LUC* reporter, both BES1-D and BES1 significantly reduced the luciferase expression than the vector control, and the effect of BES1-D was stronger (**Figure 8C**). We also performed EMSA to validate BES1 binding to the promoters of these potential target genes. BES1 can specifically bind to the promoters of *NHX2* and *CAX3 via* the E-box motif CACGTG (**Figure 8D**) but not of that of *SLAH3* and *HKT1* (**Supplementary Figure S8**). These results suggest that *NHX2* and *CAX3* genes are direct targets of BES1 in regulating plant salt tolerance, while *SLAH3* and *HKT1* are likely upregulated by an enhancement of BES1 activity in BES1-D. Consistent with these results, we found that the BES1 protein was induced at the early stage of salt treatment (3 and 6 h), with a proportional increase in the dephosphorylated active form (**Figure 8E**).

## Discussion

BR has major effects on promoting plant growth and development, and it was also involved in regulation of plant responses to environmental changes such as drought, freezing, salt, and heat stresses (Li et al., 2017; Nolan et al., 2017a; Ye et al., 2017; Nolan et al., 2019; Li et al., 2020; Albertos et al., 2022). In this study, we found that *bes1-D*, a gain-of-function mutant of BES1, showed better growth performance and lower relative electrolyte leakage than wild-type under salt stress (150 and 200 mM NaCl), while the loss-of-function mutant *BES1-RNAi* performed poorer than the wild-type plants (**Figures 1**, **2**). A consistent conclusion has been drawn by Jia et al. (2021) that *SlBZR1* cloned from tomato enhanced salt tolerance in both tomato and *Arabidopsis*. But the molecular mechanism how *SlBZR1* regulates its target genes was not clear, despite that several salt-responsive genes (i.e., *SlAREB1*, *SlDREB1*, *SlRD29, SlERF1, SlCAT2, SlAPX2*) were shown to be upregulated in *SlBZR1D*-overexpressing plants. BES1 is known to be directly regulated by BIN2 through phosphorylation (Yin et al., 2002). However, there were also evidences showing that the activation of BES1 can be mediated by PP2C phosphatases (ABI1) in a BR independent manner (Zhang et al., 2009; Wang et al., 2018; Albertos et al., 2022). These studies indicated a positive role of BZR1/BES1 in plant stress responses, and activation of BZR1/BES1 can be mediated by factors other than BIN2 only.

Transcriptome sequencing provides a full scenario of global gene expression of *bes1-D* and wild-type under salt stress (**Figure 3**). DEGs and time series clustering analyses indicated that a total of 1,170 genes were enhanced in *bes1-D* under salt stress, of which the 365 genes in Cluster 2 were specifically upregulated and the 805 genes in Cluster 3 were boosted in *bes1-D* (**Figure 4A**). Two known BES1 target genes *HSP70.4* (AT3G12580) and *HSP90.1* (AT5G52640) (Albertos et al., 2022) were observed in Cluster 2, suggesting that BES1 may directly upregulate genes in these two clusters to enhance salt tolerance. Both transcriptome and proteome analyses showed that parts of the genes involved in ABA biosynthesis, catabolism, and signaling were induced by salt stress particularly in 2-day treatment samples (**Figure 7C**). Three ABA biosynthesis genes (i.e., *ABA1*, *ABA3*, and *AAO3*) were significantly upregulated in both *bes1-D* and Col-0 under salt stress, but their salt-induced fold changes in these two materials were almost identical. Four *DREB* genes (*DREB2B*, *DREB2C*, *DREB19*, and *DREB26*) were upregulated in *bes1D_Salt_2d* (**Figure 7C**), suggesting that BES1 may activate ABA signaling under salt stress. A previous study revealed that a crosstalk of ABA and BR signaling was established through the interaction of PP2Cs and BIN2, whereby PP2Cs (ABI1 and ABI2) dephosphorylated BIN2 under normal conditions. As PP2Cs are negative regulators in ABA signaling pathway, accumulation of ABA under stress conditions will inactive PP2Cs and promote BIN2 phosphorylation thereby inhibiting BES1 and BR responsive genes (Wang et al., 2018; Jiang et al., 2019a). A novel crosstalk between ABA and BR was reported by Albertos et al. (2022) that BES1 activation mediated by PP2C phosphatases was not completely rely on BIN2. Our findings of the upregulation of *DREB* genes in *bes1-D* under salt stress provided a new clue for the crosstalk of BR and ABA signaling. Other than this, our transcriptome data demonstrated that a series of ROS related genes, particularly *GST* family, were upregulated in *bes1-D* under salt stress (**Figure 7E**). These findings are consistent with a functional study of *TaBZR2* in wheat (*Triticum aestivum*) which showed that *TaBZR2* enhances drought tolerance *via* targeting *TaGST1*, a scavenger of superoxide anions induced by drought stress (Cui et al., 2019). Expression heatmap showed that most of the anthocyanin biosynthesis genes (i.e., *PAL1, PAL2, CHS, F3H, DFR, ANS, UF3GT, PAP1*) were also upregulated in *bes1-D* under salt stress (**Figure 7D**), suggesting that BES1 promotes anthocyanins accumulation to help ROS scavenging under salt stress. A BR-induced accumulation of anthocyanins has been reported in grape (*Vitis vinifera*) (Vergara et al., 2018). It was shown that BES1 acts as an important regulator in plant response to UVB light stress. Under normal conditions, BES1 inhibits flavonoid biosynthesis genes *MYB11*, *MYB12* and *MYB111* through binding to their promoters as a transcriptional repressor. Under UVB light stress, down-regulated BES1 compromises the inhibition on these three genes and thus promoting flavonol accumulation (Liang et al., 2020). In our transcriptome data, the expression of these three genes were almost unchanged between *bes1-D* and the wild-type, either under stress or normal conditions (**Supplementary Figure S9**). This inconsistency reflected complicated regulation of flavonol accumulation in different growth periods or conditions.

In our transcriptional activation assays, BES1-D was found to regulate four ion transporters (*NHX2*, *CAX3*, *SLAH3*, and *HKT1*) possibly by binding to their promoters (**Figure 8**). Of these four genes, *CAX3* was also previously identified as putative BES1 target gene in a ChIP-chip study (Yu et al., 2011). Chromatin accessibility is highly dynamics in response to growth and environmental changes. Therefore, the targets genes of transcription factors obtained under different conditions may be different. For example, an ATAC sequencing profiling revealed that cell-type-specific differences of transcription factors in *Arabidopsis* (Maher et al., 2017). Another study of accessible chromatin regions (ACRs) in tea genome demonstrated that 226 common THSs (distal transposase hypersensitive sites) were shared by normal samples (997 THSs) and chilling treated samples (663 THSs) (Wang et al., 2021). Therefore, we speculate that BES1 may specifically regulate some of the target genes under salt stress. Indeed, our EMSA DNA-binding assays confirmed that *NHX2* and *CAX3* could specifically bind BES1.

In conclusion, our results in this study indicate that BES1-D, a constitutively active form of BES1, can increase plant salt tolerance in *Arabidopsis*. Under salt stress, the *bes1-D* mutant performed much better than the wilt-type (Col-0) and the *BES1-RNAi* line. Transcriptome sequencing results revealed that 1,170 genes were clearly upregulated in *bes1-D* compared to Col-0 under salt stress. Functional enrichment indicated that these genes were mostly enriched in key regulatory modules such as regulation of response to stress, responsive to ROS, flavonoid biosynthesis, and transmembrane transport, etc. Further proteomic analysis and qRT-PCR assays confirmed the findings from transcriptome analysis. Both transcriptomic and proteomic data confirmed that the upregulation of genes/proteins involved in regulation of response to stress, synthesis of secondary metabolites, ROS and ABA signaling, and those controlling ion transport are the key mechanisms of BES1 promotion of salt tolerance in *Arabidopsis*. Finally, transient gene transcription assays using *Arabidopsis* protoplasts and EMSA DNA binding assay confirmed that some of these genes are indeed under regulation of BES1 in plant cells.

## Data availability statement

The datasets presented in this study can be found in online repositories. Transcriptome data were deposited in NCBI Sequence Read Archive database (SRA, http://www.ncbi.nlm.nih.gov/sra) with the accession number PRJNA882207. The mass spectrometry proteomics data have been deposited to the ProteomeXchange Consortium via the PRIDE partner repository with the dataset identifier PXD036880.

## Author contributions

J-XH conceived the project; LF, Y.L., Y-LZ, G-HM, and Z-LJ performed the research and organized data; LF and J-XH wrote the manuscript; W-HL provided critical discussions and editing of the manuscript. All authors contributed to the article and approved the submitted version.
