# Supplementary material for: Integrative transcriptomic and proteomic analyses reveal a positive role of BES1 in salt tolerance in Arabidopsis
Source: Front Plant Sci. 2023 Mar 1;14:1034393. doi: 10.3389/fpls.2023.1034393 (PMC10015447; doi:10.3389/fpls.2023.1034393)
Supplement: Supplementary file 1 [file DataSheet_1.docx]

**Title: Integrated Transcriptomic and Proteomic Analyses Revealed a Positive Role of BES1 in Salt Tolerance in *Arabidopsis***

Lei Feng, Yan Li, Yu-Ling Zhou, Guang-Hua Meng, Zhao-Lin Ji, Wen-Hui Lin, Jun-Xian He


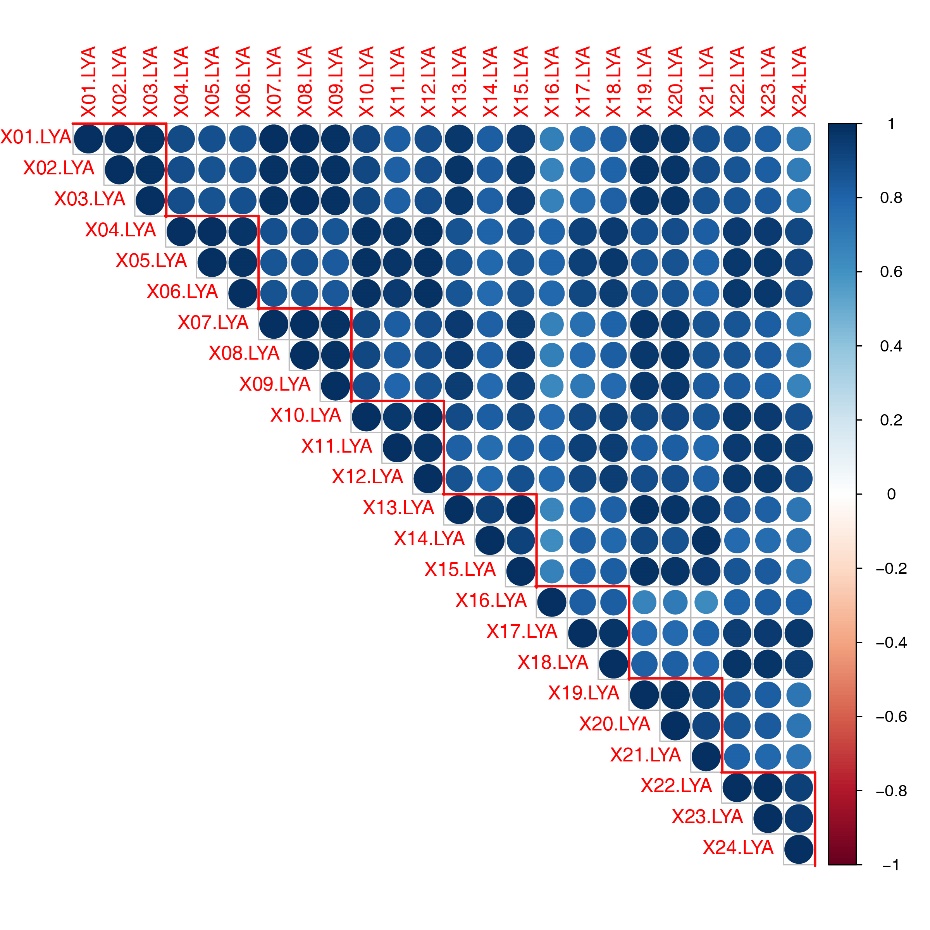


**Supplementary Figure S1.** Coefficient correlation analysis of RNA seq samples. A total of 13528 genes with average FPKM > 5 were used for this analysis. Detailed information of samples was listed in Supplementary Table S1.


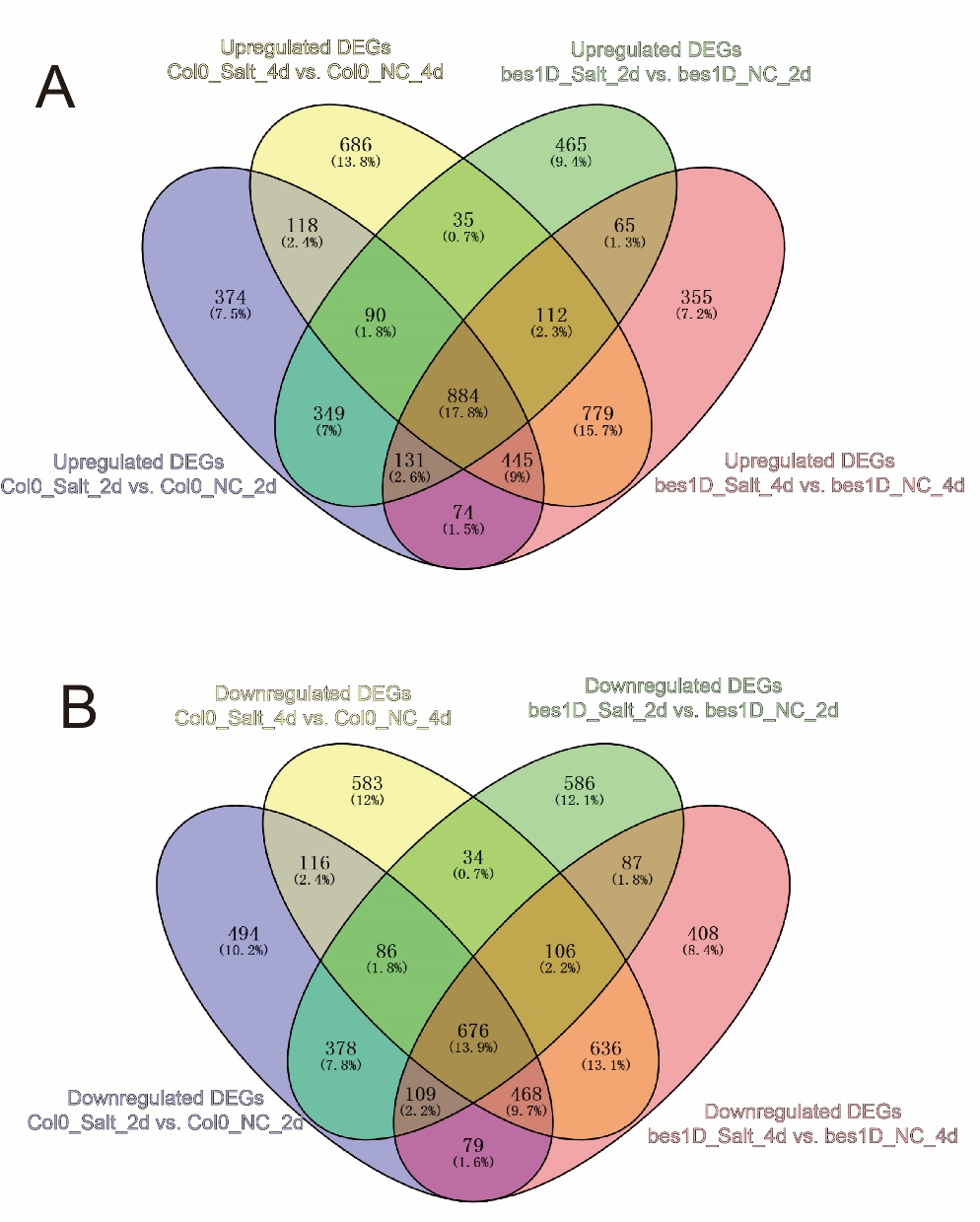


**Supplementary Figure S2.** Venn diagram showing the overlapping in different comparisons. (A) Upregulated DEGS; (B) Downregulated DEGs.


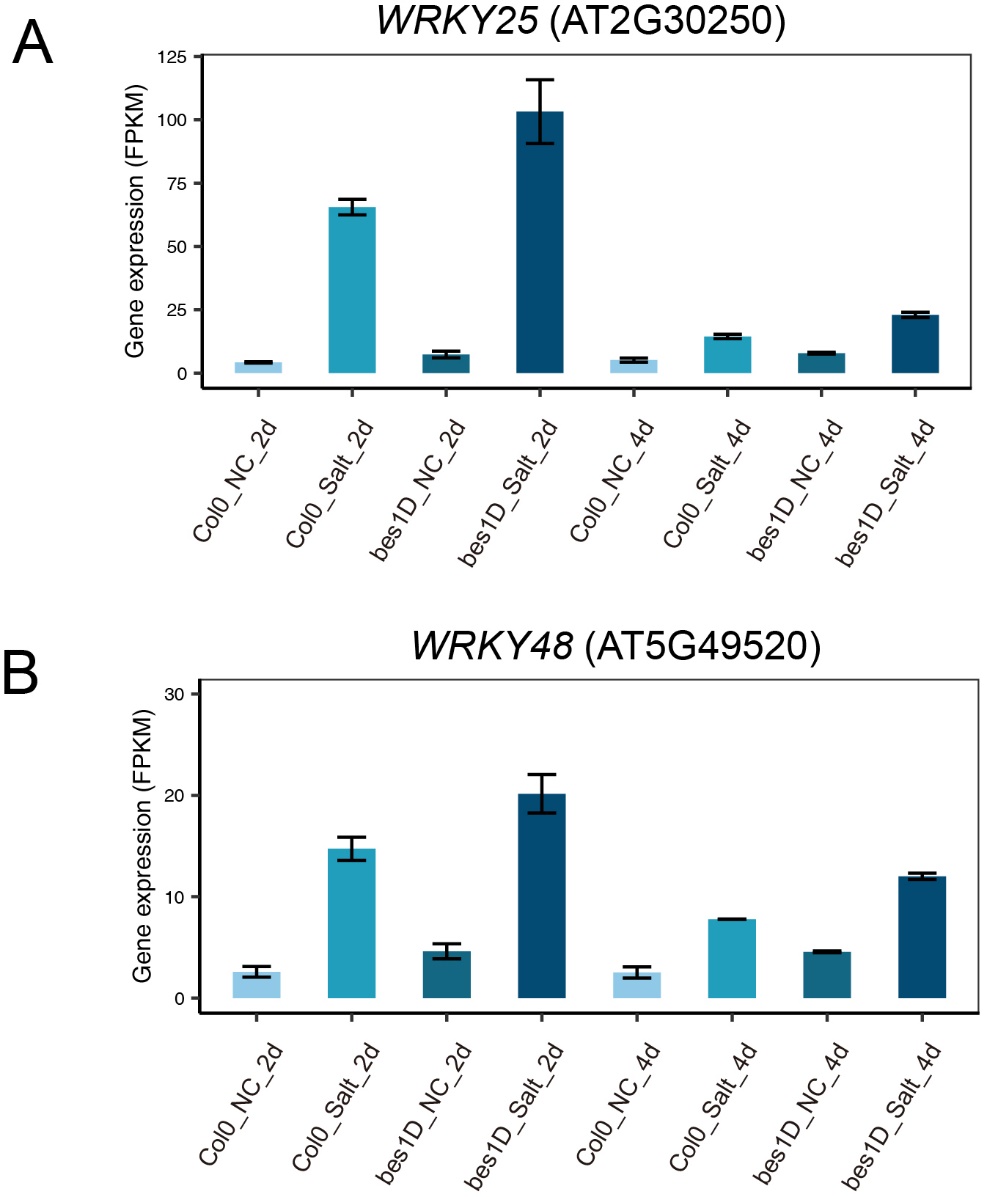


**Supplementary Figure S3.** Expression of two well-known salt-induced genes in the eight samples. (A) *WRKY25*; (B) *WRKY48*. FPKM value was inferred from transcriptome data.


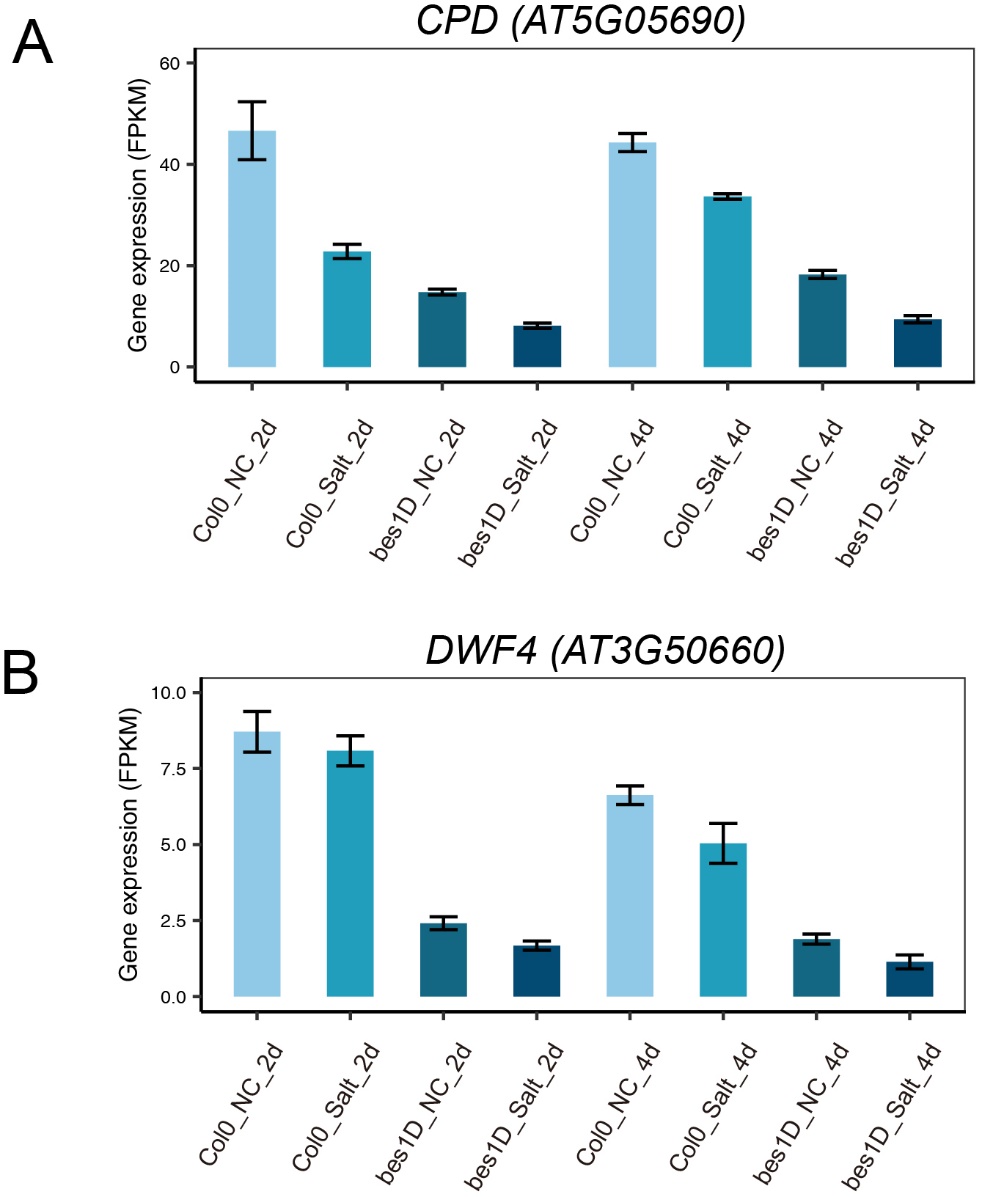


**Supplementary Figure S4.** Expression of two BR biosynthetic genes in the eight samples. (A) *CPD*; (B) *DWF4*. FPKM value was inferred from transcriptome data.


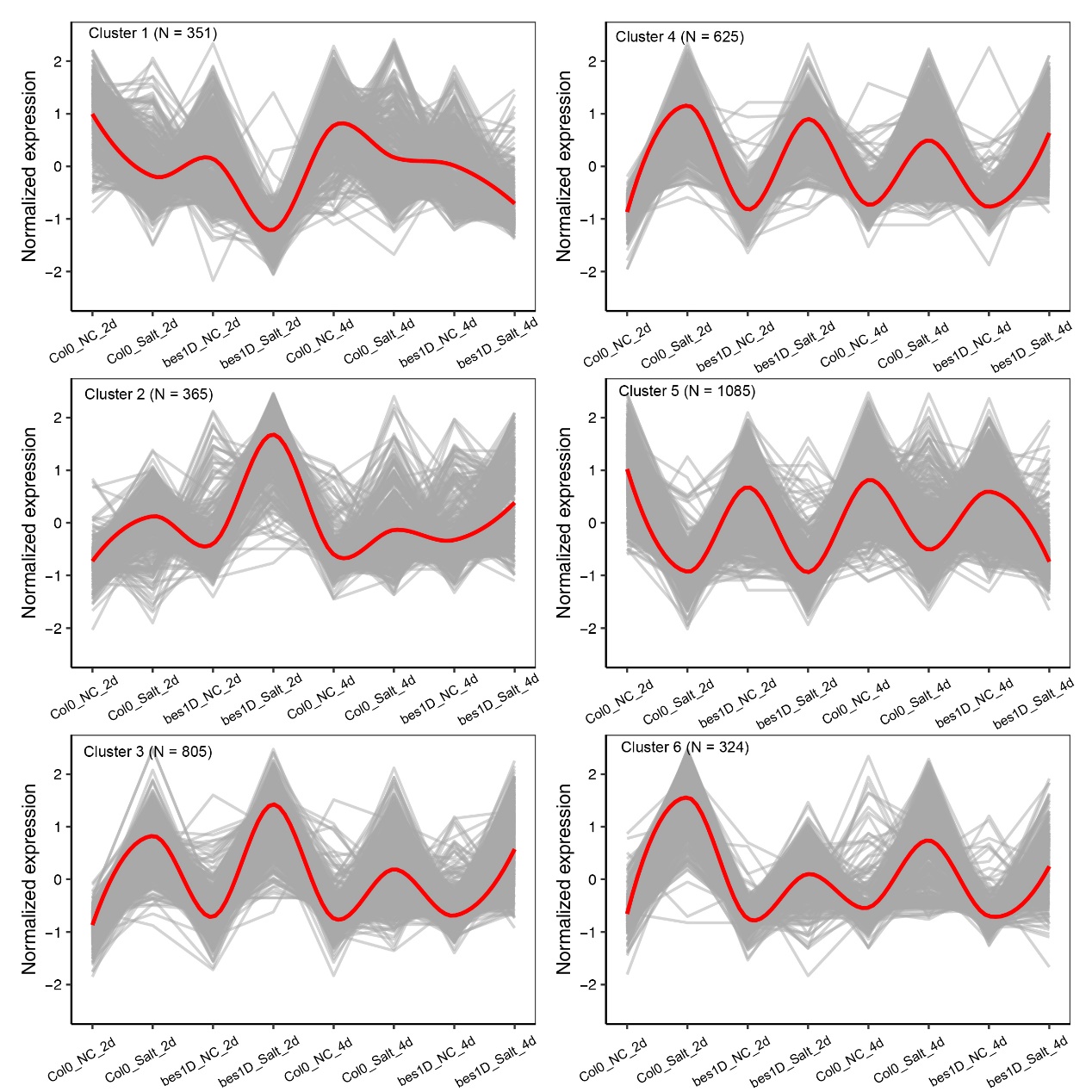


**Supplementary Figure S5.** Time Series Clustering analysis of genes in bes1-D and Col-0 under salt stress based on *Col0_NC_2d*, *Col0_Salt_2d* and *bes1D_Salt_2d*. The line plots show gene expression profiles (gray) and the mean (bold red) of the cluster.


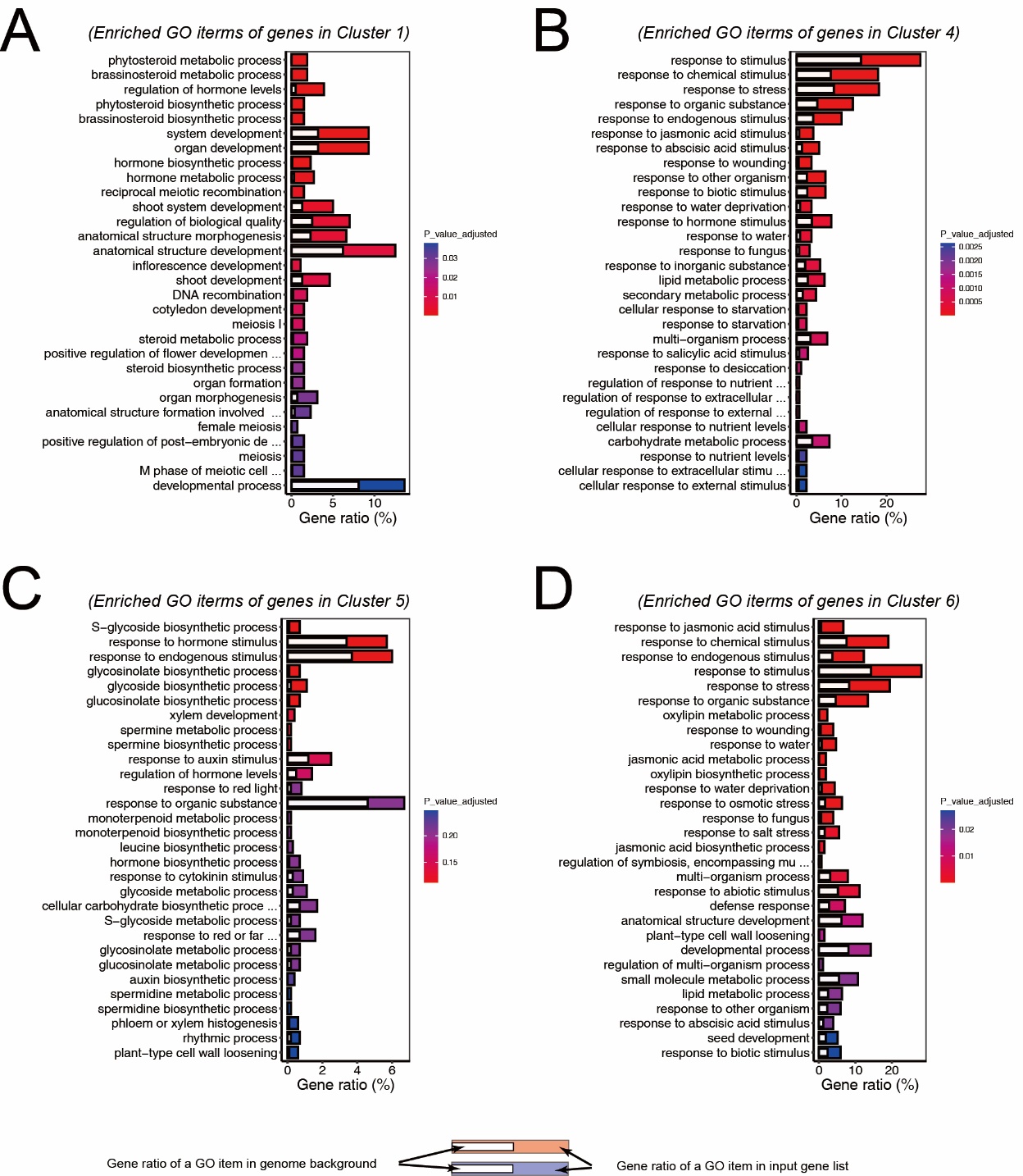


**Supplementary Figure S6.** Gene Ontology enrichment analysis for gene clusters from TCseq. (A) Cluster 1; (B) Cluster 4; (C) Cluster 5; (D) Cluster 6.


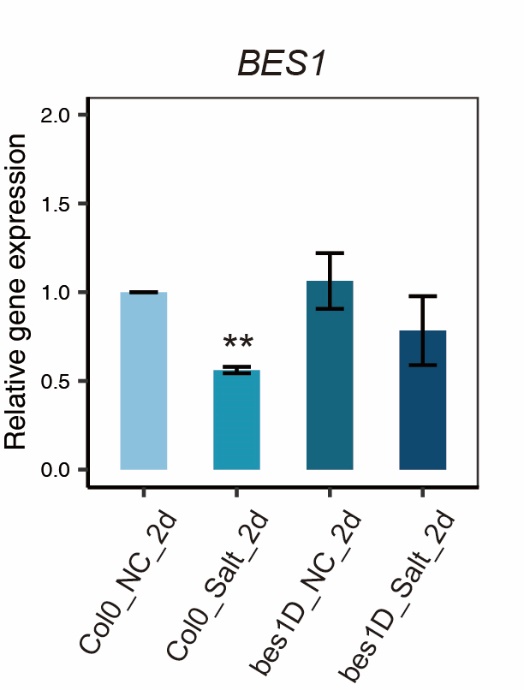


**Supplementary Figure S7.** qRT-PCR analysis of *BES1* in Col-0 and *bes1-D* under salt stress. NC, normal conditions; Salt, 150 mM of NaCl. The sample *Col-0_NC_2d* was used as control for other experimental samples. A housekeeping gene *UBQ10* (AT4G05320) was adopted as an internal reference for normalization of gene expression values. Relative expression values were calculated by using 2^(−ΔΔCt) method. Error bar means SE value of three biological repeats for each gene. Stars above error bars denote significant differences compared to *Col-0_NC_2d* (* P < 0.05, ** P < 0.01, ANOVA and Tukey’s HSD).


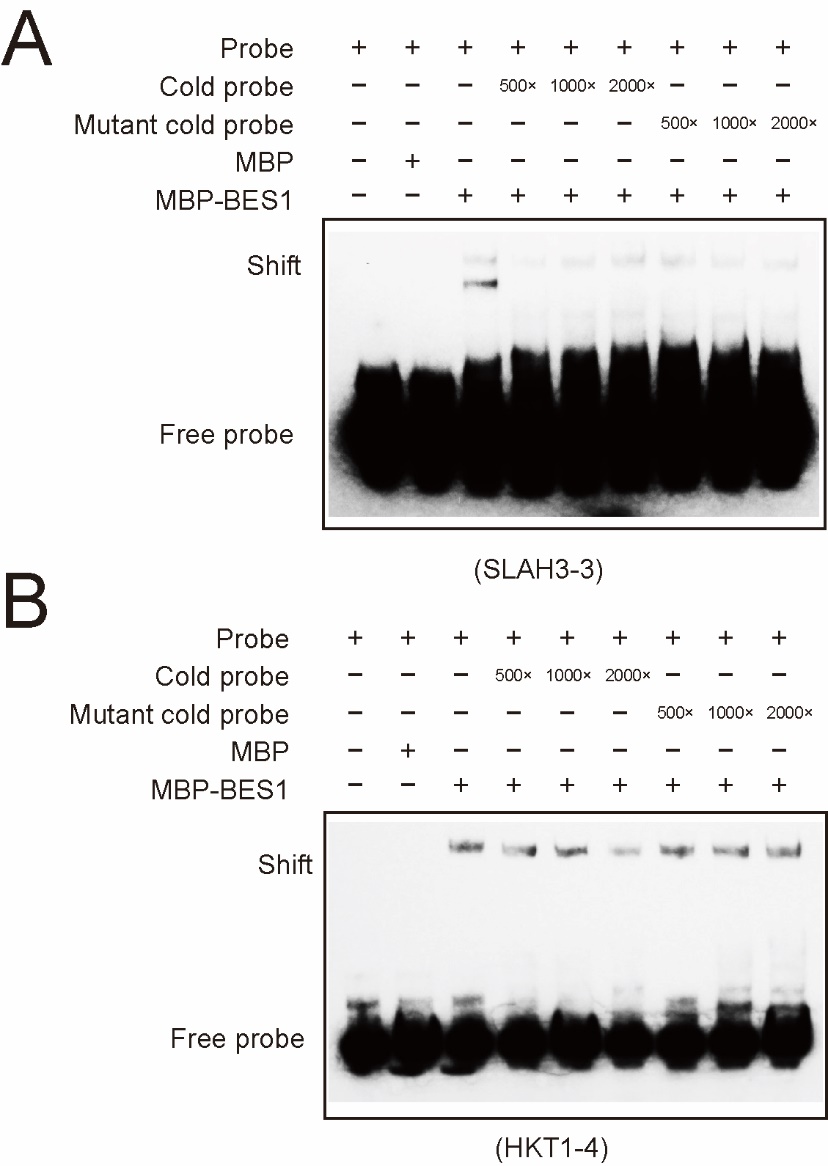


**Supplementary Figure S8.** EMSA analysis of BES1 binding to the promoter regions of SLAH3 and HKT1. Cold probes (without 3’-biotin labeling) or mutant cold probes were used to compete with the active probes for binding BES1.


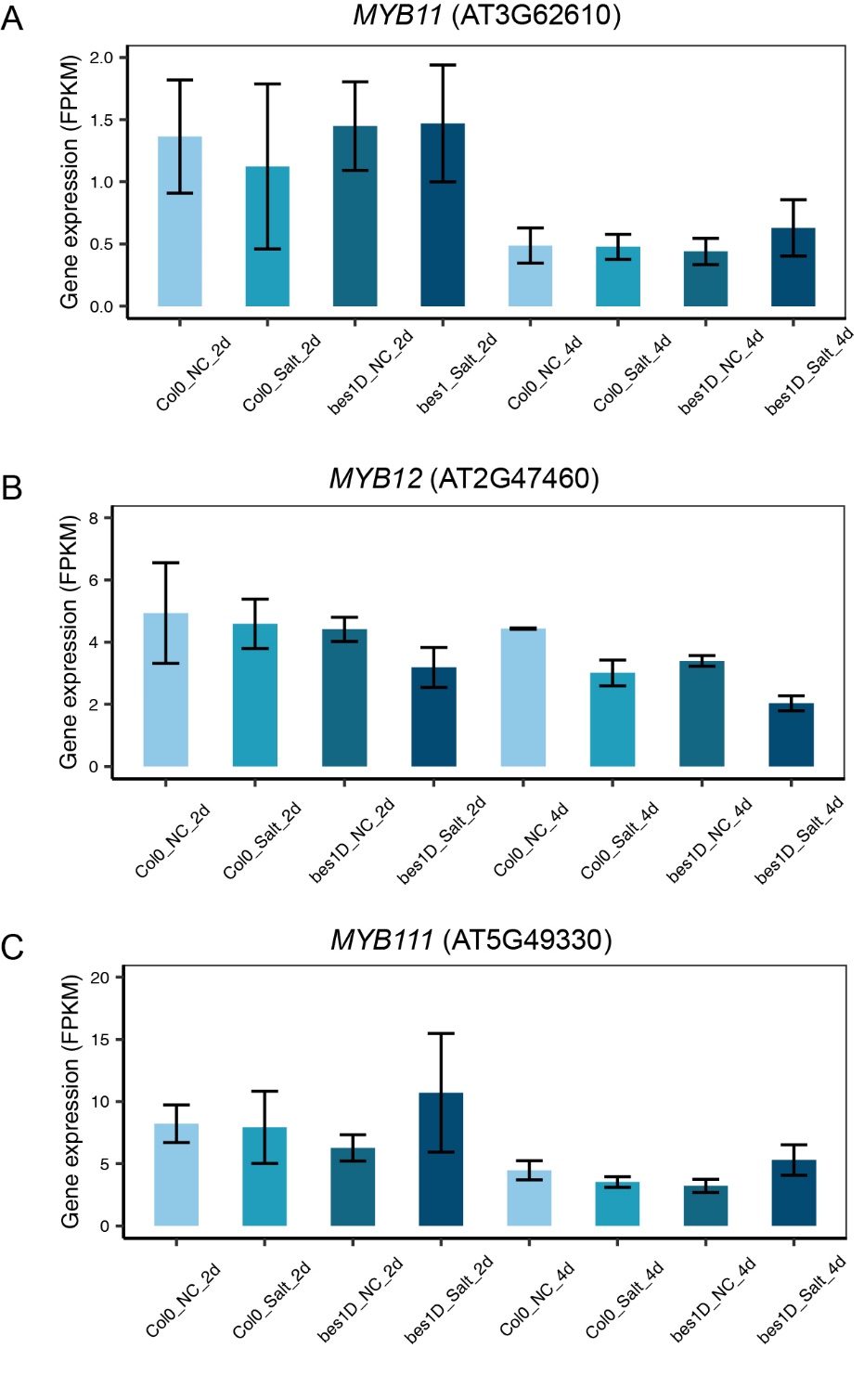


**Supplementary Figure S9.** Expression of three flavonoid biosynthesis genes in the eight samples. (A) *MYB11*; (B) *MYB12*; (C) *MYB111*. FPKM value was inferred from transcriptome data.
